# FGF2 Mediated USP42‐PPARγ Axis Activation Ameliorates Liver Oxidative Damage and Promotes Regeneration

**DOI:** 10.1002/advs.202408724

**Published:** 2025-03-17

**Authors:** Nanfei Yang, Qiang Tian, Zhenli Lei, Shuxin Wang, Nan Cheng, Zhen Wang, Xianqin Jiang, Xuqun Zheng, Wenjing Xu, Minyan Ye, Longwei Zhao, Meiyun Wen, Jianlou Niu, Weijian Sun, Pingping Shen, Zhifeng Huang, Xiaokun Li

**Affiliations:** ^1^ Oujiang Laboratory (Zhejiang Lab for Regenerative Medicine, Vision, and Brain Health) State Key Laboratory of Macromolecular Drugs and Large‐scale Preparation School of Pharmaceutical Sciences Wenzhou Medical University Wenzhou Zhejiang 325035 China; ^2^ Department of Colorectal Surgery The First Affiliated Hospital of Wenzhou Medical University Wenzhou 325027 China; ^3^ State Key Laboratory of Pharmaceutical Biotechnology and Clinical Stem Cell Center The Affiliated Drum Tower Hospital of Nanjing University Medical School School of Life Sciences Nanjing University Nanjing 210023 China; ^4^ School of Integrative Medicine Nanjing University of Chinese Medicine Nanjing 210023 China; ^5^ Department of Pharmacology School of Basic Medical Sciences Wenzhou Medical University Wenzhou Zhejiang 325035 China

**Keywords:** FGF2, liver regeneration, nano‐therapy, PPARγ, redox balance, USP42, ubiquitination

## Abstract

Liver regeneration is critical for maintaining whole‐body homeostasis, especially under exposure to deadly chemical toxins. Understanding the molecular mechanisms underlying liver repair is critical for the development of intervention strategies to treat liver diseases. In this study, ubiquitin‐specific Proteases 42 (USP42) is identified as a novel deubiquitinases (DUB) of peroxisome proliferators‐activated receptor γ (PPARγ) in hepatocytes. This DUB interacted, deubiquitinated, and stabilized PPARγ, and increased PPARγ targeted proliferative and antioxidative gene expressions, which protects the liver from carbon tetrachloride (CCL4) induced oxidative injury and promotes liver regeneration. In addition, fibroblast growth factor 2 (FGF2) initiated USP42 expression and enhanced the interaction between USP42 and PPARγ during the liver regeneration process. Moreover, the PPARγ full agonist, rosiglitazone (RSG), possesses the ability to further reinforce the USP42‐PPARγ interplay, which enlightens to construct of an extracellular vesicle‐based targeting strategy to activate the liver USP42‐PPARγ axis and promote liver regeneration. In summary, the work uncovers the importance of USP42‐PPARγ axis‐mediated liver tissue homeostasis and provides a promising regimen to target this protein‐protein interplay for liver regeneration.

## Graphical abstract

USP42 is identified as a novel DUB of PPARγ in hepatocytes. USP42 mediated PPARγ deubiquitylation determines its transcriptional preference on proliferative and redox balance genes. USP42 knockdown exacerbates liver damage and delays regeneration. FGF2 is the upstream signal that initiates and activates the USP42‐PPARγ axis. Targeting the USP42‐PPARγ axis using EV@FGF2‐RSG efficiently protects against damage and promotes liver repair.

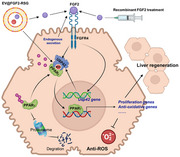

## 1 Introduction

The liver plays a central role in whole‐body homeostasis by regulating various physiological functions such as metabolism, detoxification of drugs, and bile transport.^[^ ^1^ ^]^ When encountering different damaging stressors such as surgery, chemical toxins, alcohol, and viruses, hepatocytes in liver tissue die, resulting in partial loss of liver function and reduction of liver mass.^[^ ^2^ ^]^ To maintain the physiological balance, the liver has a unique regenerative capacity where hepatocytes can initiate proliferation to compensate for the loss or death of parenchymal cells until the liver mass and morphology are fully restored.^[^ ^3^ ^]^ However, in certain conditions such as chronic liver diseases or aging, the regenerative capacity of the liver is significantly impaired, which may lead to liver dysfunction, jeopardizing the body's health and even posing a risk of death.^[^ ^4^ ^]^ Several studies have attempted to establish therapeutic methods for promoting liver regeneration, including the use of cytokines,^[^ ^5^ ^]^ growth factors,^[^ ^6^ ^]^ antibodies,^[^ ^7^ ^]^ specific agonists/antagonists,^[^ ^8^ ^]^ and cell therapy interventions.^[^ ^9^ ^]^ These evidences highlight the critical importance of understanding and elucidating the molecular mechanisms underlying liver regeneration for its application in the treatment of liver diseases.

As a master metabolic regulator, PPARγ participates in regulating glucose and fat, as well as oxidative‐reduction (redox) metabolism, which maintains the physiological function of hepatocytes.^[^ ^10^ ^]^ The full agonists of PPARγ, thiazolidinediones (such as rosiglitazone), have been successfully used in the treatment of metabolic and inflammatory liver diseases.^[^ ^11^ ^]^ Moreover, the liver‐specific PPARγ‐null mice exhibited impaired regeneration phenomenon in the setting of diet‐induced chronic steatosis, suggesting that PPARγ is a key regulator in complete liver regeneration.^[^ ^12^ ^]^ Our previous work also indicates that PPARγ T166 phosphorylation mediates the aging‐ or obesity‐induced liver injury and repair.^[^ ^13^ ^]^ However, the detailed molecular mechanism of how PPARγ regulates liver repair has not yet been fully elucidated. In fact, as a crucial signaling sensor, PPARγ can precisely perceive various stimuli under physiological or pathological conditions, and alter its molecular behavior.^[^ ^14^ ^]^ Nevertheless, the upstream signals that activate PPARγ to regulate liver regeneration are still poorly understood.

Fibroblast Growth Factors (FGFs) are the important signaling molecules that regulate liver regeneration and serve as key mitogens for controlling hepatocyte proliferation.^[^ ^3^, ^15^ ^]^ Abnormalities in FGF/FGFR‐related signaling functions can significantly affect liver cell division, hindering liver regeneration and repair.^[^ ^16^ ^]^ Some FGFs (such as FGF1 and FGF15), have been reported to promote hepatocyte rejuvenation and liver regeneration. For example, FGF1 stimulates DNA synthesis in rat liver cells. FGF1 is produced by hepatocytes during liver regeneration and has mitogenic effects on endothelial cells and hepatic stellate cells;^[^ ^17^ ^]^ FGF15 (its human homolog: FGF19), produced by intestinal cells, interacts with liver FGFR4‐β‐Klotho, downregulating the first enzyme involved in bile acid synthesis, CYP7A1.^[^ ^18^ ^]^ FGF15 deficiency leads to a significant increase in intrahepatic bile acid levels, attenuates liver regeneration, and increases mortality after partial hepatectomy.^[^ ^19^ ^]^ Yet, the FGF/FGFR downstream signaling cascade remains largely reclusive.

Ubiquitination is a fundamental process in molecular biology that involves the attachment of a small protein called ubiquitin to target proteins.^[^ ^20^ ^]^ This process plays a crucial role in regulating various cellular processes, including protein degradation, DNA repair, cell cycle control, and signal transduction.^[^ ^21^ ^]^ Ubiquitin‐protein conjugates formed through ubiquitination can be recognized by the proteasome, which is a large protein complex responsible for degrading unwanted or damaged proteins. Once recognized, the ubiquitinated proteins are targeted for degradation by the proteasome, ultimately leading to their breakdown into smaller peptides.^[^ ^22^ ^]^ Dysregulation of ubiquitination and deubiquitylation is closely associated with liver damage and tissue repair. For instance, Ubiquitin‐Specific Proteases 21 (USP21)‐mediated deubiquitylation of histone H2A promotes hepatocyte regeneration.^[^ ^23^ ^]^ SIRT2‐mediated deacetylation and deubiquitylation of C/EBPβ attenuates the liver injury and accelerate live repair.^[^ ^24^ ^]^ To date, the role of PPARγ ubiquitination modulation in liver function has not yet been reported.

Here, based on our previously constructed transgenic mice, we successfully identified ubiquitin‐specific proteases 42 (USP42) as a novel DUB of PPARγ. Considering that cases of liver damage caused by global organic pollutant contamination are becoming predominant,^[^ ^25^ ^]^ we used the carbon tetrachloride (CCL4) model to study the function of USP42 in chemical toxins‐induced liver injury in this study. As per our findings, USP42 is able to interact with PPARγ, serves as an “eraser” to remove the ubiquitination modification, and maintains the stability of PPARγ protein. This effect influences the classical transcriptional behavior of PPARγ on hepatocyte proliferative and anti‐oxidative damage genes, thus regulating liver damage protection and regeneration. More importantly, the USP42‐PPARγ axis can be activated by FGF2, which further guided us to establish a new extracellular vesicle‐based nanomedical strategy to target liver USP42‐PPARγ interaction and promote liver regeneration.

## 2 Results

### 2.1 Identification of USP42 is a Novel DUB for PPARγ in Hepatocytes

In our previous study, we identified the T166 phosphorylation pattern in the PPARγ DNA binding domain (DBD), and utilizing the global T166A (alanine mutation, TA) mutant mouse strains, to mimic the persistent in vivo dephosphorylation status, we confirmed the biological function of this phosphorylation in beige adipocyte differentiation.^[^ ^13^ ^]^ Here, we explored the function of the T166 mutant in liver tissue and found that the TA mutant protected mice from the carbon tetrachloride (CCL4)‐induced liver injury (Figure S1A, Supporting Information). Several studies use the expression of proliferating cell nuclear antigen (PCNA) as a key marker for liver repair, as it is an intracellular nuclear protein that plays a critical role in DNA synthesis and repair as well as in promoting hepatocyte proliferation^[^ ^1^, ^26^ ^]^; thus, we measured PCNA levels to reflect the liver's repair capacity. The result showed that TA mutant liver tissues exhibited higher levels of proliferating cell nuclear antigen (PCNA) (Figure S1B, Supporting Information). Interestingly, when analyzing PPARγ gene expression and its protein content, we found that the mRNA levels between WT and TA were similar (Figure S1C, Supporting Information), but the TA protein abundance was significantly higher than that in WT counterparts (Figure S1D, Supporting Information), which suggested that the TA mutant stabilized PPARγ protein. To confirm the in vivo observations, we overexpressed wild‐type (WT) and T166A (TA) mutant PPARγ2 (60kDa) in AML‐12 cells, a type of PPARγ‐null hepatocyte cell line. Protein content analysis revealed that TA significantly increased the PPARγ protein levels (Figure S1E, Supporting Information). Moreover, TA accelerated the proliferation of AML‐12 cells (Figure S1F, Supporting Information), which suggested that TA induced the PPARγ protein stabilization and was involved in the regulation of hepatocyte proliferation and liver repair.

Next, to dig out what factors participated in the PPARγ protein stabilization, WT, and TA mutants were overexpressed as the probes in AML‐12 cells. Then, by using a specific anti‐PPARγ antibody (Santa Cruz Biotech. sc‐7273) to perform the co‐immunoprecipitation assay (Co‐IP) assay, PPARγ and its interacted proteins were affinity purified and enriched. The protein complexes were subjected to mass spectrometry (MS/MS) to identify co‐purifying proteins (Figure S2A, Supporting Information). Parallelly, the mouse IgG‐purified proteins were used as the isotype control group. Trough excluding the IgG‐purified proteins and merging the hits in every repeat, we identified 98 interacting proteins in the WT group and 117 interacting proteins in the TA group. To identify high‐confidence protein‐protein interactions (PPIs), we employed the MiST algorithm (mass spectrometry interaction statistics) to quantitatively score protein‐protein interactions (PPIs) (Table S1, Supplementary Table1). As a result, 85 proteins were determined as TA‐specific PPIs. Among these, only one DUB, USP42, was identified (Figure S2B, Supporting Information). Co‐IP assay demonstrated that TA indeed enhanced interaction between PPARγ and USP42 (Figure S2C, Supporting Information). Thus, we speculated that USP42 was able to protect PPARγ protein from degradation, and participated in liver regeneration.

**Figure 1:**
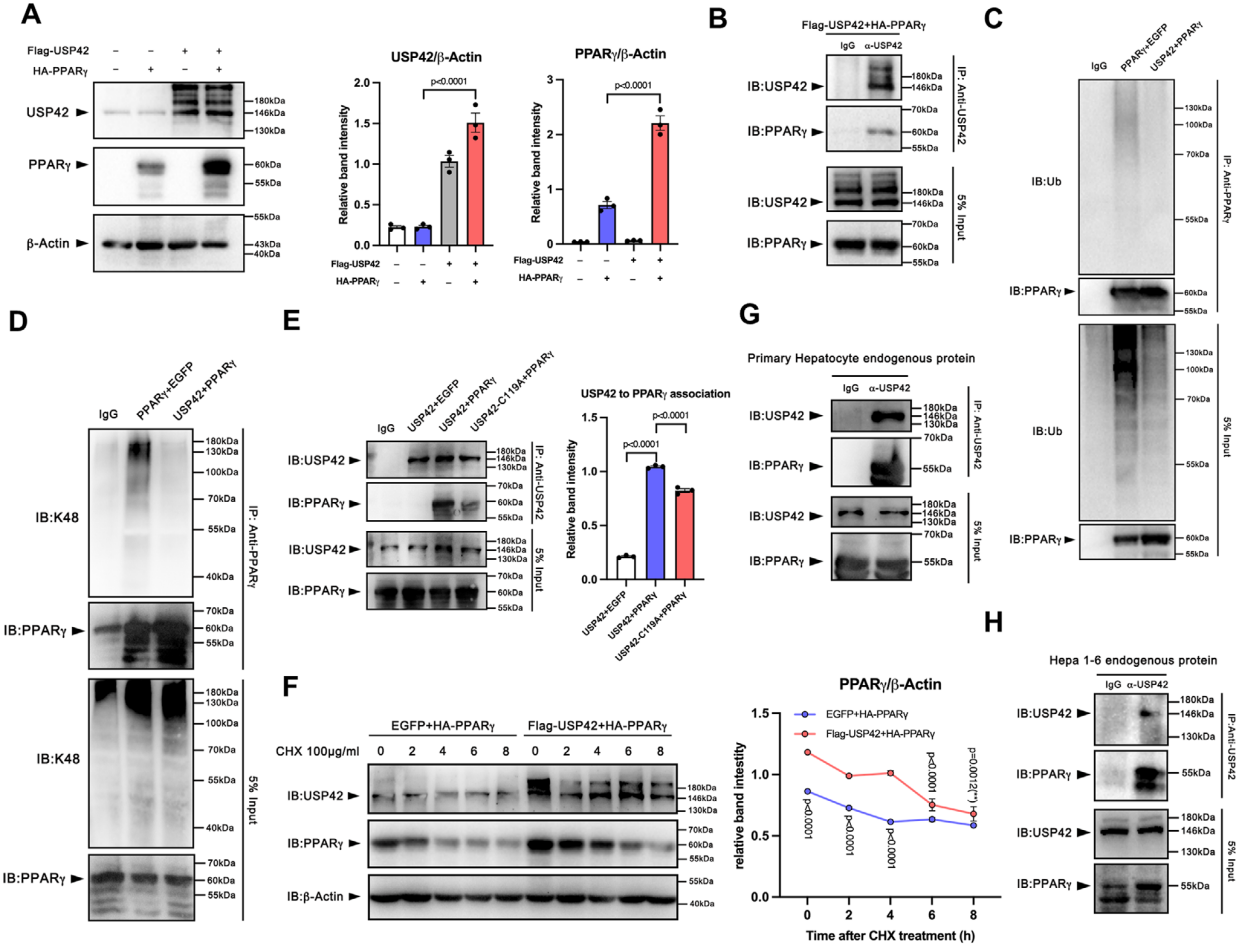
USP42 interacts, deubiquitinates, and stabilizes PPARγ. A) HEK293T cells were transfected with the WT PPARγ, USP42 plasmid, or co‐transfected with the two plasmids. After 24 h transfection, total cell lysis was applied to western blotting to analyze the protein levels of PPARγ and USP42. Experiments were repeated three times. B) HEK293T cells were transfected with WT PPARγ and USP42 plasmids. Co‐immunoprecipitation (Co‐IP) analysis of interaction between two proteins. Experiments were repeated three times. C) Ubiquitination assay of PPARγ in HEK293T cells co‐transfected with WT PPARγ, EGFP, and USP42 and treated with 10 µM MG‐132 for 12 h. D) K48‐linked ubiquitination assay of PPARγ in HEK293T cells co‐transfected with WT PPARγ, EGFP, and USP42 and treated with 10 µM MG‐132 for 12 h. E) HEK293T cells were transfected with WT PPARγ and WT USP42 or C119A USP42 plasmids. Co‐immunoprecipitation (Co‐IP) analysis of interaction between PPARγ and USP42. Experiments were repeated three times. F) Stability analysis of PPARγ protein in HEK293T cells. G, H) Co‐immunoprecipitation (Co‐IP) analysis of the endogenous interplay of PPARγ and USP42 in primary hepatocytes (G) and Hepa1‐6 cell line (H). Experiments were repeated three times. Data were analyzed by one‐way ANOVA followed by Tukey's test (A, E) or two‐way ANOVA followed by Bonferroni's test (F). Data are presented as mean ± SEM. ^＊^ *p* < 0.05. ^＊＊^ *p* < 0.01, ^＊＊＊^ *p* < 0.001.

We then examined whether USP42 was associated with PPARγ. To distinguish the ectopic overexpressed and endogenous PPARγ, in 293T cells, we co‐transfection of USP42 and PPARγ2 (60kDa), and found that USP42 markedly increased the protein level of PPARγ (**Figure** **1**A). The PPI between ectopically expressed USP42 and PPARγ was verified by Co‐IP (Figure 1B). Meanwhile, an immunoprecipitation (IP)–based ubiquitination assay was further conducted, and the result provided evidence that USP42 decreased the ubiquitination level of PPARγ (Figure 1C). Next, to study the type of polyubiquitin chains on PPARγ by USP42, we used specific anti‐K48 and anti‐K63 antibodies to detect the ubiquitination, and the data demonstrated that exogenously transduced USP42 significantly reduced K48‐linked ubiquitination of PPARγ (Figure 1D; Figure S2D, Supporting Information). Previous studies have demonstrated that mouse USP42 cysteine 119 sites (as cysteine 120 sites in human USP42) is the key residue controlling the catalytical activity of USP42.^[^ ^27^ ^]^ To further study the catalytical correlation between USP42 and PPARγ, we ectopically expressed the catalytically inactive mutant USP42 C119A (cysteine mutated into alanine) and PPARγ in 293T cells and analyzed their interaction. As shown in Figure 1E and Figure S2E (Supporting Information), the C119A mutant inhibited the binding of USP42 to PPARγ, and correspondingly damaged the deubiquitylation effect of WT USP42 on PPARγ. Furthermore, we found that when USP42 was overexpressed, the degradation rate of PPARγ was dramatically decreased (Figure 1F). Finally, to investigate whether this newly identified PPI occurred in hepatocytes, we selected two cell models— Hepa1‐6 cell line and primary hepatocyte to verify endogenous interaction mode. In both cell models, the anti‐USP42 antibody was able to enrich the endogenous protein, and the PPARγ protein was also detected in the Co‐IP (Figure 1G). To confirm this endogenous interaction, we established the siRNA system to knockdown the endogenous USP42. As shown in Figure S2F (Supporting Information), the siRNA‐4 sequence exhibited the most powerful capacity to knock down the USP42 protein, and the mRNA knockdown efficiency was verified (Figure S2G, Supporting Information). We then used this siRNA to interfere with USP42 expression and tested the USP42‐PPARγ interaction. The results showed that in both Hepa1‐6 cells and primary hepatocytes, the total ubiquitylation level of PPARγ was enhanced upon the USP42 knockdown (Figure S2H,I, Supporting Information). Interestingly, when we analyzed the USP42 expression using hepatocellular carcinoma (HCC) cDNA chip from 26 human samples, we found that USP42 was highly expressed in the tumor area compared to that in the adjacent tissue (Figure S2J, Supporting Information). Data from the TCGA database also indicated a correlation between higher USP42 expression and low survival rate (Figure S2K, Supporting Information). These data indirectly corroborated that the USP42 expression is associated with hepatocyte proliferation. Taken together, these results demonstrated that USP42 is a novel DUB for PPARγ protein stabilization.

### 2.2 USP42 Expression is Correlates with the Liver Regeneration

**Figure 2:**
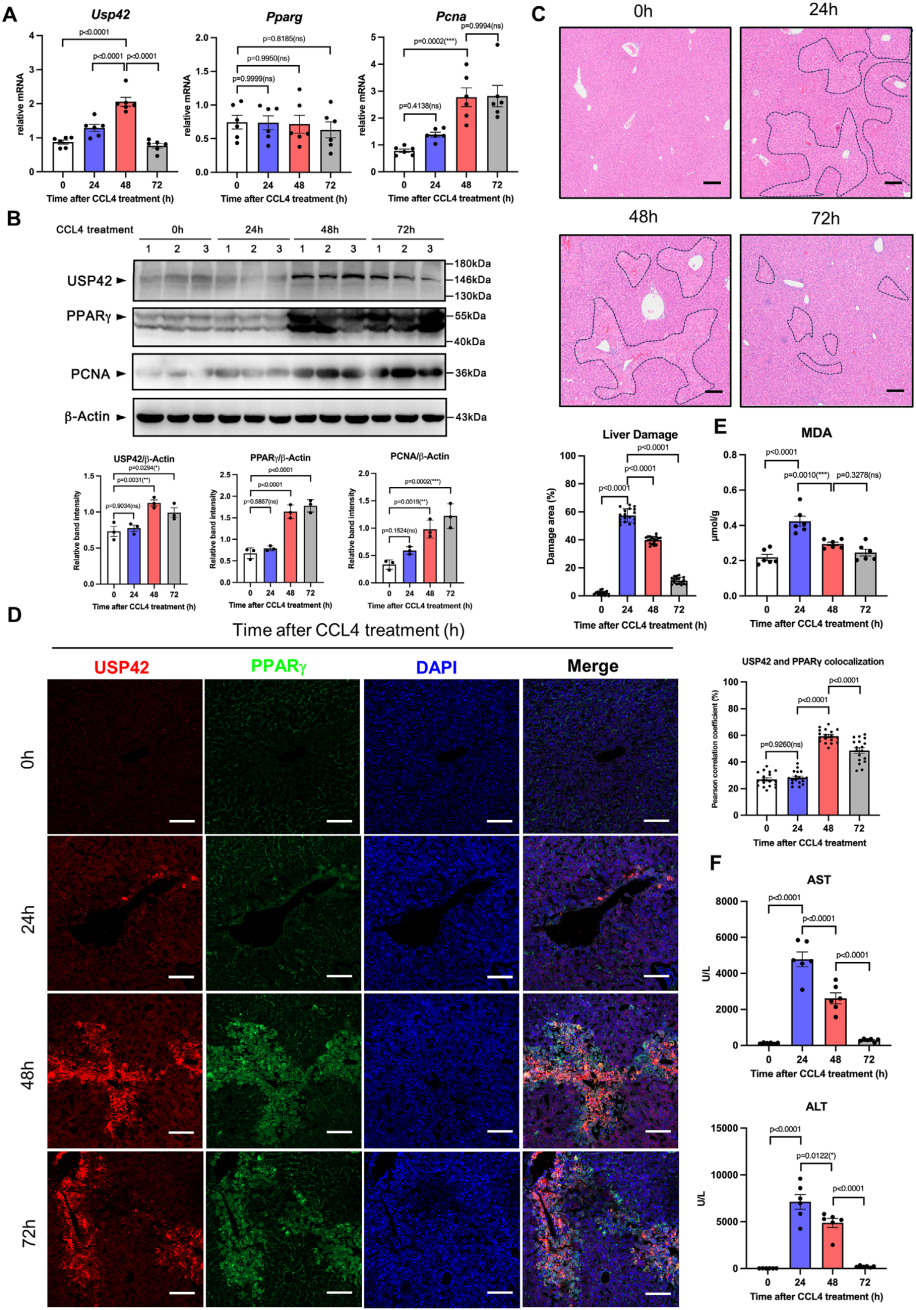
USP42 and PPARγ protein levels are correlated with liver regeneration state. A) Q‐PCR analysis of the mRNA levels of USP42, PPARγ, and PCNA in liver tissues from male C57BL/6J mice at indicated times (hours) following CCL4 treatment (*n* = 6 in every group). B) Isolated liver tissues were homogenized. The whole tissue protein was analyzed by western blotting to evaluate the levels of USP42, PPARγ, and PCNA (*n* = 6). Every lane contains the total protein from two mice. Every point in the statistical chart represents the band signal intensity of one lane. C) H&E staining of liver tissues (*n* = 6). 100 × magnification, scale bar, 100 µm. Each H&E slide is captured in three fields of view, and the liver injury area in each field of view is calculated to determine the average injury area. Every point in the statistical chart represents the injury area percentage in each field of view. D) Confocal microscopy analysis of the co‐localization of USP42 and PPARγ protein in liver tissues (*n* = 6). 200 × magnification, scale bar, 100 µm. Each slide is captured in three fields of view, and the Pearson correlation coefficient was calculated. Very point in the statistical chart represents the Pearson correlation coefficient in each field of view. E) The MDA content in the liver tissue was measured using a commercial kit *n* = 6). F) The activity of ALT and AST was measured by using the fully automated dry biochemical analyzer NX700i (FUJIFILM) (*n* = 6). Data were analyzed by one‐way ANOVA followed by Tukey's test (A‐F). Data are presented as mean ± SEM. ^＊^ *p* < 0.05. ^＊＊^ *p* < 0.01, ^＊＊＊^ *p* < 0.001.

We used a CCL4‐induced acute liver injury mouse model to study the potential role of USP42 in liver injury and regeneration and evaluated USP42 levels and PPARγ stability in detail in liver tissue. The mice were intraperitoneally injected with CCL4. At specific time points (24, 48, and 72 h) after treatment, the liver tissues were collected for analysis. At 24 h after CCL4 treatment, we observed severe liver damage, including hepatocyte destruction, vacuolization, and lipid accumulation (**Figure** **2**C; Figure S3C, Supporting Information). At 48 h after treatment, the damage area was decreased. The PCNA mRNA and protein levels were dramatically upregulated, suggesting that regeneration was initiated in the liver tissues (Figure 2A–C; Figure S3A, Supporting Information). At 72 h after treatment, the liver tissue was spontaneously repaired, and the high PCNA mRNA/protein expression levels remained unchanged (Figure 2A–C).

Accompanying the process from damage to regeneration, the liver injury markers, serum alanine aminotransferase (ALT), and aspartate aminotransferase (AST) activities were sharply increased at 24 h, but gradually decreased from 24–72 h after CCL4 treatment (Figure 2F). Previous studies have demonstrated the redox state controls liver injury and regeneration.^[^ ^28^ ^]^ Thus, we further evaluated the malondialdehyde (MDA) and hydrogen peroxide (H_2_O_2_) levels in the liver tissues, which are markers of ROS stress. The results showed that both MDA and H_2_O_2_ levels were markedly elevated at 24 h and started to decrease upon the initiation of regeneration (Figure 2E; Figure S3B, Supporting Information). In contrast to the ROS state, antioxidative factors, including reduced glutathione (GSH) and superoxide dismutase (SOD), were highly enriched at 48 h, suggesting that hepatocytes started to generate antioxidants to combat damage and promote tissue repair (Figure S3B, Supporting Information). Similarly, by quantifying the mRNA and protein content of USP42, we found that USP42 gene expression was significantly upregulated at 48 h and then returned to the basal state at 72 h (Figure 2A). However, higher USP42 protein levels were maintained during the regeneration phase (48–72 h), compared to 24 h. The PPARγ protein content was significantly upregulated during liver regeneration (Figure 2B), whereas the gene expression levels were unchanged (Figure 2A). Next, by using the immunofluorescent staining assay, we confirmed that USP42 and PPARγ were mainly co‐localized at 48 and 72 h time points (Figure 2D). Together, these data indicate that USP42 and PPARγ protein levels were positively correlated with liver regeneration. The USP42‐PPARγ axis may be involved in the regulation of liver regeneration.

### 2.3 USP42‐PPARγ Axis Inhibition Exacerbates Liver Damage and Delays Liver Regeneration

**Figure 3:**
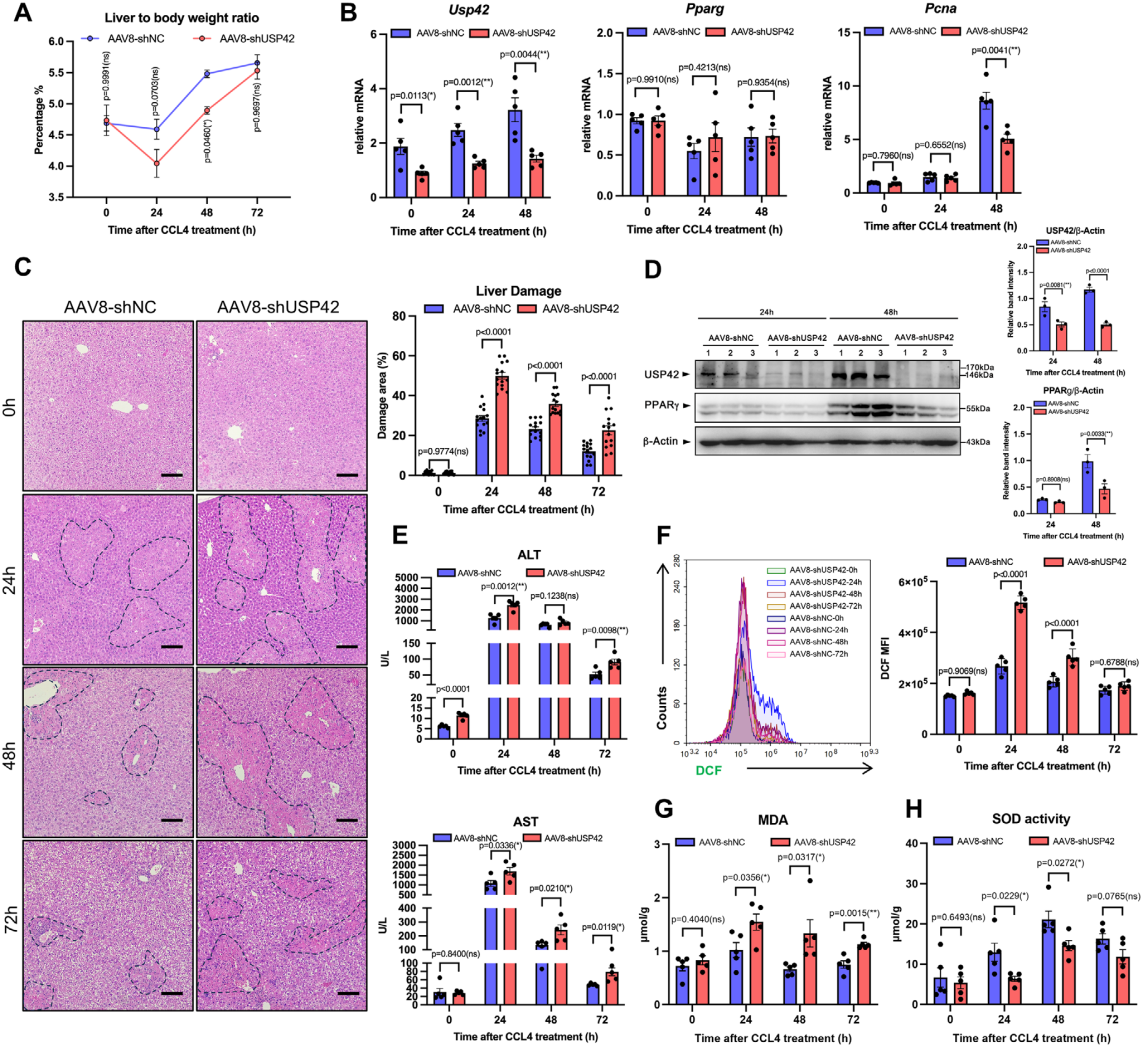
USP42 knockdown exacerbates liver damage and increases ROS during liver regeneration. Male C57BL/6J mice were injected with AAV8‐shNC or AAV8‐shUSP42 via the tail vein. Three weeks after AAV8 treatment, the mice were subjected to CCL4 treatment. At the indicated time point, the mice were euthanasia, and the serum and liver tissues were applied to the experiments. A) Ratio of liver‐to‐body weight of AAV8‐shNC or AAV8‐shUSP42 group on the indicated time after CCL4 treatment (*n* = 5). B) Q‐PCR analysis of the mRNA levels of USP42, PPARγ, and PCNA in liver tissues (*n* = 5). C) H&E staining of liver tissues (*n* = 5). 100 × magnification, scale bar, 100µm. Each H&E slide is captured in three fields of view, and the liver injury area in each field of view is calculated to determine the average injury area. Every point in the statistical chart represents the injury area percentage in each field of view. D) Isolated liver tissues were homogenized. The whole tissue protein was analyzed by western blotting to evaluate the levels of USP42 and PPARγ (*n* = 5). Every lane contains total protein from one or two mice. Every point in the statistical chart represents the band signal intensity of one lane. E) The activity of ALT and AST was measured by using the fully automated dry biochemical analyzer NX700i (FUJIFILM) (*n* = 5). F) The liver tissues were digested into cell suspension and incubated with DCFH‐DA for 30 min. The fluorescence intensity was detected using flow cytometry (*n* = 5). G, H) The MDA content (G) and the SOD activity (H) in the liver tissue was measured using the commercial kits (*n* = 5). Data were analyzed by two‐way ANOVA followed by Bonferroni's test (A‐H). Data are presented as mean ± SEM. ^＊^ *p* < 0.05. ^＊＊^ *p* < 0.01, ^＊＊＊^ *p* < 0.001.

To further study the regulatory role of USP42 in liver regeneration, we generated high titer adeno‐associated virus carrying an expressing cassette for shRNA against USP42 (AAV8‐shUSP42) or for scrambled control shRNA (AAV8‐shNC), and injected the viruses into C57BL/6J mice via the tail vein. As expected, AAV8‐shUSP42 deceased gene expression of USP42 under the basal condition (Figure S4A, Supporting Information). Next, we challenged the mice with CCL4 treatment and analyzed the mRNA and protein levels of USP42 and PPARγ. At the mRNA level, AAV8‐shUSP42 significantly inhibited the mRNA expression of USP42 but had no effect on PPARγ gene expression (**Figure** **3**B). At the protein level, we observed a significant decrease in USP42 expression after AAV8‐shUSP42 treatment (Figure 3D). At 24 h after CCL4 treatment, the PPARγ protein levels in the AAV8‐shUSP42 group were similar to those in the AAV8‐shNC group. However, at the 48 h time point (regeneration program initiation), the USP42 knockdown significantly attenuated the PPARγ protein content (Figure 3D).

In line with the USP42 expression levels, CCL4‐induced liver injury, as indicated by the necrotic area observed on histological hematoxylin and eosin (H&E) staining and serum ALT/AST levels, was more pronounced in AAV8‐shUSP42 treated mice than in AAV8‐shNC controls (Figure 3C,E). To evaluate the effect of USP42 knockdown on ROS level, we used the ROS fluorescent probe 2,7‐dichloro‐dihydro‐fluorescein diacetate (DCFH‐DA) to measure the intracellular ROS in total liver cells. DCFH‐DA itself is non‐fluorescent and can freely pass through the cell membrane, and intracellular ROS can oxidize it to generate fluorescent DCF. The result showed that AAV8‐shUSP42 treatment exhibited significantly higher ROS levels at 24 and 48 time points measured (Figure 3F). Moreover, the MDA levels followed the same trend (Figure 3G). To study the role of USP42 in the regulation of antioxidative factors, the SOD activity and the GSH level were analyzed. As shown in Figure 3H, the USP42 knockdown markedly reduced the total SOD activity in liver tissues. Total GSH, as well as the reduced GSH and GSSG levels, were also decreased after AAV8‐shUSP42 treatment (Figure S4E–G, Supporting Information). To analyze hepatocyte apoptosis, we used the TUNEL staining to test the DNA breakage in tissue sections. The results revealed that AAV8‐shUSP42 increased the DNA damage in the hepatocytes (Figure S4H, Supporting Information). Next, we evaluated the anti‐oxidative gene expression status in relation to the USP42 levels. Previous studies had identified several antioxidant enzymes, such as proteins in the SOD and glutathione peroxidase (Gpx) families, and the transcriptional factor Nrf2, were the direct target genes of PPARγ. ^[^ ^29^ ^]^ Therefore, we isolated the primary hepatocytes and cultured in vitro. USP42 siRNA was transfected into the cells, followed by CCL4 treatment. Q‐PCR screening of SOD and Gpx families revealed that CCL4 treatment enhanced the SOD3, Gpx3, and Gpx4 gene expression in the siNC group. However, the siUSP42 transfection significantly inhibited the CCL4‐induced gene expressions of SOD3, Gpx3, and Gpx4 (Figure S4I,J, Supporting Information). Cellular ROS levels increased upon the USP42 knockdown using the DCF and MitoSox probes (Figure S4K–M, Supporting Information). Collectively, these data indicated that USP42 knockdown exacerbates liver oxidative damage.

Simultaneously, the USP42 function in liver regeneration was examined. Because of compensatory hyperplasia and hypertrophy of hepatocytes during liver regeneration,^[^ ^3^ ^]^ we analyzed the liver weight. Under the AAV8‐shUSP42 treatment, the body weight of the mice did not change; however, the USP42 knockdown caused a weight reduction in the liver tissue (Figure S4B,C, Supporting Information). Consequently, the liver‐to‐body weight ratio decreased (Figure 3A). Our findings suggest that USP42 knockdown may disturb overall liver repair. By analyzing the H&E‐stained sections, we noticed that the rate of damage area decline was slower in the USP42 knockdown group (Figure 3C), indirectly suggesting that USP42 is critical for liver regeneration. To confirm this, PCNA mRNA levels were quantified using Q‐PCR, and the results showed that PCNA gene expression was remarkably enhanced at 48 h. However, when USP42 was knocked down, PCNA mRNA expression was significantly reduced (Figure 3B). PCNA protein staining indicated that USP42 knockdown decreased PCNA‐positive cells and fluorescence intensity (Figure S4D, Supporting Information). Taken together, these data suggest that USP42 plays a key role in regulating hepatocyte proliferation and liver regeneration.

To further confirm the regulatory role of the USP42‐PPARγ axis in liver repair, we used a specific PPARγ antagonist, GW9662, to inhibit PPARγ transcriptional activity in the presence of high USP42 expression 48 h after CCL4 treatment (Figure S5A, Supporting Information). The ALT/AST assay showed that PPARγ inhibition significantly increased the serum AST/ALT levels, indicating exacerbated liver damage (Figure S5B, Supporting Information). Correspondingly, H&E staining of liver sections showed that GW9662 treatment markedly increased the area of liver damage and delayed liver repair (Figure S5C, Supporting Information). Subsequent analysis of the liver's redox state revealed that inhibition of PPARγ activity significantly downregulated the total SOD activity and GSH levels while increasing the MDA content (Figure S5D–F, Supporting Information). Gene expression analysis confirmed that GW9662 inhibited *Sod3*, *Gpx3*, and *Gpx4* expression (Figure S5D–F, Supporting Information). Finally, we used confocal microscopy to analyze the protein expression of PCNA in the liver tissue and its colocalization with the hepatocyte marker HNF4α. As shown in Figure S5H,I (Supporting Information), GW9662 significantly inhibited PCNA protein expression in hepatocytes. In summary, these data show that even under conditions of high USP42 expression, the inhibition of PPARγ activity can significantly hinder liver self‐regeneration. This further supports the critical role of the USP42‐PPARγ signaling axis in liver repair.

### 2.4 USP42‐PPARγ Axis Controls the Redox Balance and Proliferation Activity in a Hepatocyte Model

**Figure 4:**
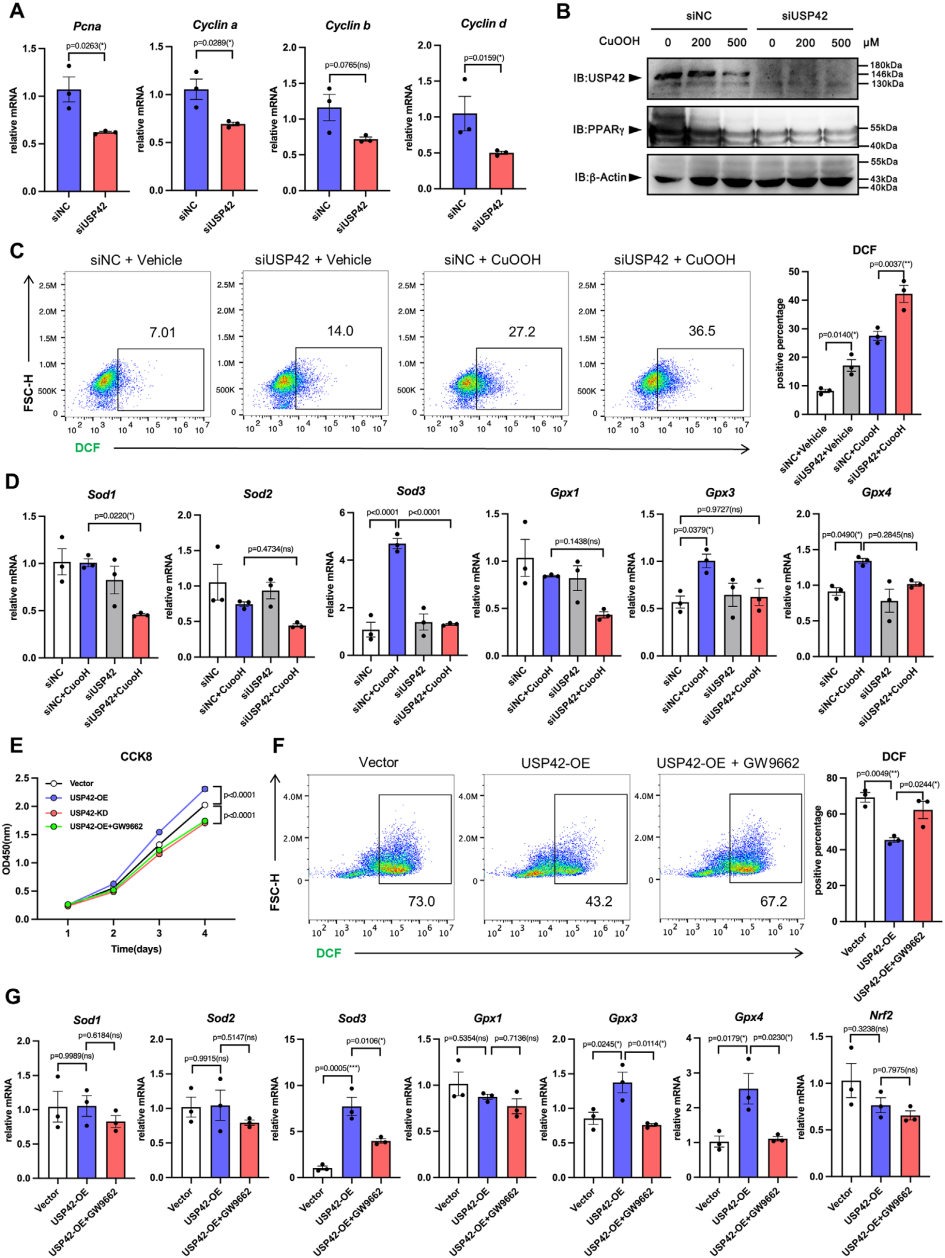
The USP42‐PPARγ axis regulates hepatocyte proliferation and redox balance. A) Hepa1‐6 cells were transfected with siNC or siUSP42 small interfering RNA. After 48 h transfection, the cells were collected and homogenized in Trizol regent. Q‐PCR analysis of the mRNA levels of PCNA, Cyclin A, Cyclin B, and Cyclin D in Hepa1‐6 cells (*n* = 3). B) Hepa1‐6 cells were transfected with siNC or siUSP42 small interfering RNA. After 48 h transfection, the cells were treated with 0, 200, or 500 µM CuOOH for 4 h. Western blotting analysis of the protein level of USP42 and PPARγ. C) The siNC and siUSP42 transfected Hepa1‐6 cells were subjected to 500 µM CuOOH treatment for 4h. The total ROS level was evaluated by the commercial kit and flow cytometry (*n* = 3). D) The siNC and siUSP42 transfected Hepa1‐6 cells were subjected to 500 µM CuOOH treatment for 4h. Q‐PCR analysis the mRNA levels of SOD1, SOD2, SOD3, GPX1, GPX3 and GPX4 (*n* = 3). E) Hepa1‐6 USP42 overexpressed cell (USP42‐OE) and USP42 knockdown cell (USP42‐KD) were treated with saline or GW9662. The growth curve was measured by CCK‐8 assay (n = 3). F) USP42‐OE cell and control cell (Vector) were treated with saline or GW9662 upon the 500 µM CuOOH administration. The total ROS level was evaluated by the commercial kit and flow cytometry (*n* = 3). G) USP42‐OE cell and control cell (Vector) were treated with saline or GW9662 upon the 500 µM CuOOH administration. Q‐PCR analysis the mRNA levels of SOD1, SOD2, SOD3, GPX1, GPX3, GPX4 and Nrf2 (*n* = 3). Data were analyzed by one‐way ANOVA followed by Tukey's test (C, D, F, and G) or two‐way ANOVA followed by Bonferroni's test (E) or Student's t‐test (A). Data are presented as mean ± SEM. **^＊^** *p* < 0.05. **^＊＊^** *p* < 0.01, **^＊＊＊^** *p* < 0.001.

We established a cell line model to further confirm our results in liver tissues. Hepa1‐6 cells were treated with cumene hydroperoxide (CuOOH), an organic oxidizing agent, to induce ROS burst. CuOOH (500 µM) significantly decreased USP42 protein levels, accompanied by PPARγ protein reduction (**Figure** **4**B). Using this model, we found that USP42 knockdown enhanced the CuOOH‐induced increase in the total ROS levels (Figure 4C). Similar to the findings in primary hepatocytes, *Sod3*, *Gpx3*, and *Gpx4* gene expression was inhibited by siUSP42 transfection (Figure 4D). These findings suggested that USP42 is a protective factor against ROS‐induced hepatocyte damage. Simultaneously, USP42 knockdown in Hepa1‐6 cells inhibited the expression of proliferative genes including *Pcna*, *Cyclin a*, *Cyclin b*, and *Cyclin c* (Figure 4A).

Next, to investigate the mechanism by which the USP42‐PPARγ axis controls the expression of antioxidative and proliferative genes, we constructed the USP42 overexpressed (USP42‐OE) and the USP42 knockdown (USP42‐KD) stable Hepa1‐6 cell lines. Using the CCK8 assay, CFSE, and EdU staining methods, we tested the cell line proliferative activity. USP42‐OE cells proliferated faster than vector controls, whereas USP42‐KD cells exhibited the opposite effect. Interestingly, when GW9662 was used to treat USP42‐OE cells, their enhanced proliferation was blocked (Figure 4E; Figure S6A,B, Supporting Information), suggesting that PPARγ transcription is essential for USP42‐mediated pro‐proliferation function. Gene expression analysis indicated that PPARγ antagonism significantly hindered the USP42 overexpression‐induced upregulation of *Pcna*, *Cyclin a*, *Cyclin b*, and *Cyclin c* (Figure S6C, Supporting Information). These data reveal that the USP42‐PPARγ axis determines the Hepa1‐6 cell proliferation. In the cell model, the effect of the USP42‐PPARγ axis on redox balance was also studied. We used the DCF probe to analyze total ROS in Hepa1‐6 cells. As shown in Figure 4C, USP42 knockdown by siRNA transfection enhanced CuOOH‐induced ROS upregulation in Hepa1‐6 cells. In contrast, USP42 overexpression decreased the ROS levels after CuOOH treatment. However, GW9662 partially abolished USP42 overexpression‐induced ROS attenuation (Figure 4F). In addition, USP42‐OE cells exhibited higher gene expression of *Sod3*, *Gpx3*, and *Gpx4*; however, PPARγ antagonism effectively blocked this beneficial effect (Figure 4G). To determine how USP42 protects the liver from oxidative damage, we evaluated the effects of DUB on PPARγ transcriptional activity. ChIP‒qPCR analysis indicated that USP42‐OE preferentially induced PPARγ to bind to the promoters of *Pcna*, *Sod3*, *Gpx3*, and *Gpx4* genes, whereas GW9662 treatment inhibited this effect (Figure S6D, Supporting Information). Taken together, these results show that the USP42‐PPARγ axis controls redox balance and proliferation activity in hepatocytes.

### 2.5 FGF2 is An Activator for the USP42‐PPARγ Axis

The results described in the previous section raised the important question of whether the signaling factor upregulated USP42 levels and activated the USP42‐PPARγ axis during liver regeneration. Previous studies have shown that FGFs secreted by hepatocytes play a crucial role in mediating liver repair.^[^ ^30^ ^]^ Therefore, we speculated that FGFs activate the USP42‐PPARγ axis. To verify this hypothesis, we first collected liver tissue from Vehicle and CCL4‐treated mice and screened FGF family gene expression using Q‐PCR. As shown in Figure S7A (Supporting Information), several FGFs were upregulated by CCL4, including FGF2, FGF5, and FGF23. In contrast, the expression levels of FGF4 and FGF8 were decreased. Among these, FGF2 showed the greatest upregulation of gene expression. Protein‐level analysis confirmed that FGF2 expression was significantly upregulated (Figure S7B, Supporting Information). Next, we examined the effects of FGF2 on USP42 expression. In the Hepa1‐6 cell model, we found that FGF2 treatment significantly increased USP42 mRNA levels but had no effect on PPARγ mRNA expression (Figure S7C, Supporting Information). To further determine whether FGF2 enhanced USP42‐PPARγ interaction, we performed Co‐IP in 293T and Hepa1‐6 cell models. In 293T cells, USP42 and PPARγ were overexpressed, followed by stimulation with FGF2. After 36 h of treatment, FGF2 increased PPARγ protein levels (Figure S7D, Supporting Information), and markedly reinforced the interaction between USP42 and PPARγ (Figure S7E, Supporting Information). In Hepa1‐6 cells, endogenous USP42‐PPARγ interplay was also significantly enhanced by FGF2 (Figure S7F, Supporting Information), and the ubiquitination level of PPARγ was simultaneously attenuated (Figure S7G, Supporting Information). Since FGFR4 is the major FGF receptor expressed in hepatocytes, we tested the impact of FGFR4 inhibition on the USP42‐PPARγ interaction. We used roblitinib (Rob), a selective blocker of FGFR4 signaling, to treat Hepa1‐6 cells. Co‐IP results indicated that Rob administration effectively inhibited the association of USP42 with PPARγ (Figure S7H, Supporting Information).

To further verify the activation role of FGF2 in the USP42‐PPARγ axis, an FGF2 neutralizing antibody was used to block FGF2 signaling in cell and animal models. As shown in Figure S8A, FGF2 antibody treatment markedly decreased USP42 and PPARγ protein levels. Gene expression analysis and ubiquitination detection experiments indicated that FGF2 reduced USP42 protein levels by inhibiting USP42 gene expression (Figure S8B, Supporting Information), whereas its effect on reducing PPARγ protein levels was mediated by enhancing PPARγ ubiquitination and degradation (Figure S8C, Supporting Information). Correspondingly, treatment with the FGF2 antibody dramatically reduced the expression of antioxidant and proliferative genes in Hepa 1–6 cells (Figure S8D,E, Supporting Information). CuOOH stimulation also significantly increased intracellular ROS levels (Figure S8F, Supporting Information). These data indicate that endogenously secreted FGF2 activates the USP42‐PPARγ axis. Similar conclusions were drawn from the animal models, where treatment with an FGF2‐neutralizing antibody significantly increased oxidative damage in the liver and inhibited its repair (Figure S8G–J, Supporting Information). Collectively, these results suggest that FGF2 is an activator of the USP42‐PPARγ axis.

### 2.6 FGF2‐Mediated Liver Protection and Regeneration Effect is Impeded by USP42 Knockdown

**Figure 5:**
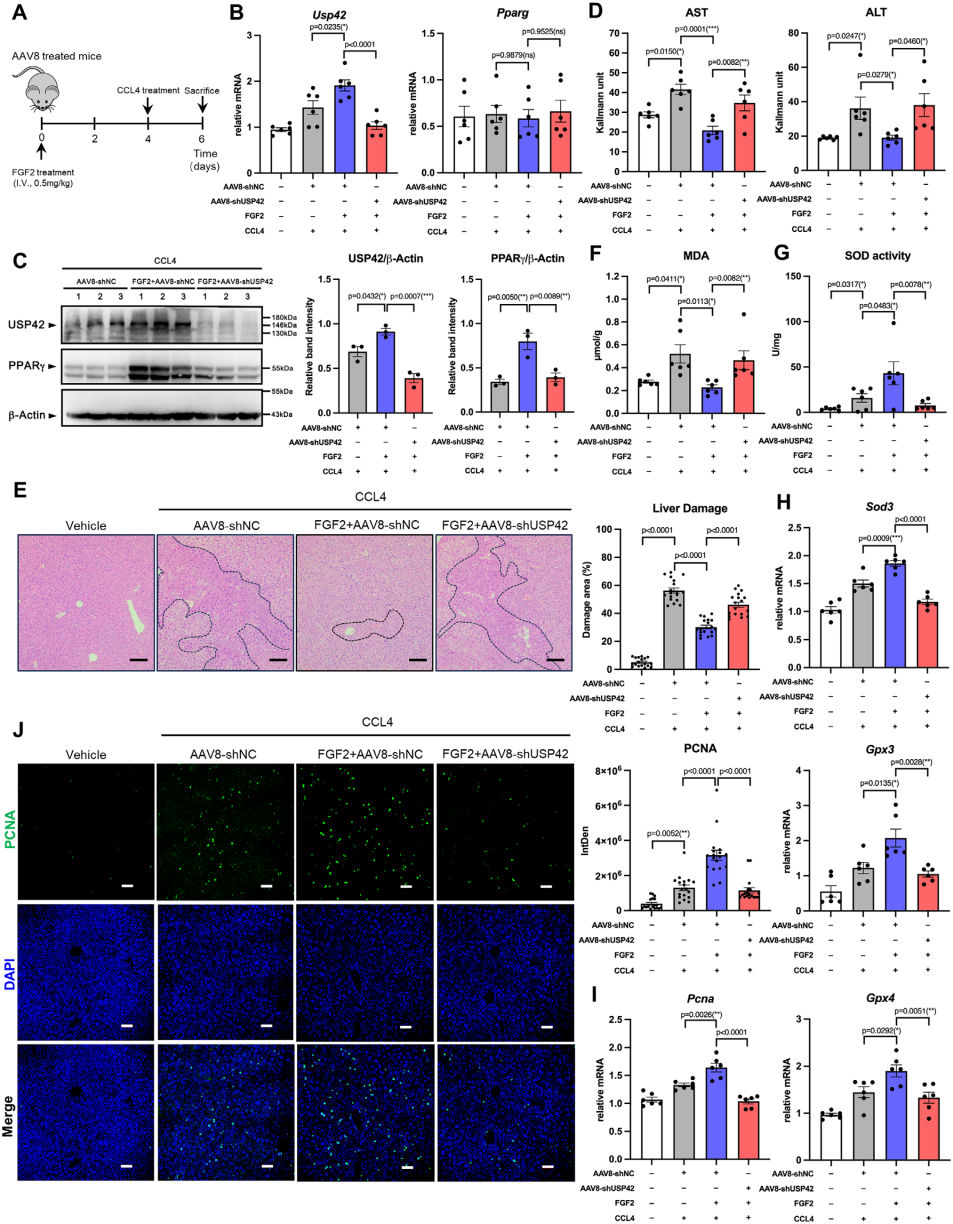
USP42 knockdown inhibits FGF2‐induced liver regeneration. A) Schematic of the experiment. Male C57BL/6 mice were pre‐treated with AAV8‐shNC or AAV8‐shUSP42 for 3 weeks. Then, mice were pre‐treated with 0.5 mg kg^−1^ FGF2 every two days. Following the FGF2 treatment, the mice were subjected to CCL4 treatment, and after 48 h, the mice were euthanasia, and the serum and liver tissues were applied to the experiments. B) Q‐PCR analysis of the mRNA levels of USP42 and PPARγ in liver tissues (*n* = 6). C) Western blotting analysis of USP42 and PPARγ protein levels in liver tissues (*n* = 6). Every lane contains the total protein from two mice. Every point in the statistical chart represents the band signal intensity of one lane. D) The activity of ALT and AST was measured by using the commercial kits (*n* = 6). E) H&E staining of liver tissues (*n* = 6). 100 × magnification, scale bar, 100µm. Each H&E slide is captured in three fields of view, and the liver injury area in each field of view is calculated to determine the average injury area. Every point in the statistical chart represents the injury area percentage in each field of view. F, G) The MDA content (F) and the SOD activity (G) in the liver tissue was measured using the commercial kits (*n* = 6). H) Q‐PCR analysis of the mRNA levels of SOD3, GPX3, and GPX4 in liver tissues (*n* = 6). I) Q‐PCR analysis of the mRNA level of PCNA in liver tissues (*n* = 6). J) Confocal microscopy analysis of PCNA protein‐positive cells in liver tissues (*n* = 6). 200× magnification, scale bar, 50µm. Each slide is captured in three fields of view, and the integrated density (IntDen) was measured by Image J software. Every point in the statistical chart represents the IntDen value in each field of view. Data were analyzed by one‐way ANOVA followed by Tukey's test (B‐J). Data are presented as mean ± SEM. **^＊^** *p* < 0.05. **^＊＊^** *p* < 0.01, **^＊＊＊^** *p* < 0.001.

To confirm that USP42 is a key node in FGF2‐mediated liver repair, we used AAV8‐shUSP42 to knock down USP42 and then injected FGF2 into mice. AAV8‐shNC was used as a control. At 48 h after CCL4 treatment, liver tissue and serum were collected for subsequent analyses (**Figure** **5**A). We quantified USP42 levels in the liver tissues and found that FGF2 induced USP42 mRNA transcription and increased USP42 protein levels (Figure 5B,C). For PPARγ, FGF2 had no effect on PPARγ mRNA expression but significantly enhanced its protein content. Upon the AAV8‐shUSP42 treatment, PPARγ protein levels markedly decreased (Figure 5B,C). These data indicate that the USP42‐PPARγ axis in the liver was regulated by FGF2 in vivo. Serum ALT/AST quantification showed that FGF2 significantly decreased the ALT and AST levels in AAV8‐shNC‐treated mice. However, by comparing FGF2‐treated AAV8‐shNC and AAV8‐shUSP42 groups, we found that USP42 knockdown inhibits FGF2‐mediated protection of the liver (Figure 5D). Histological H&E staining confirmed this observation (Figure 5E). Consistent with the ALT/AST trends, FGF2 treatment notably decreased MDA levels in liver tissue; however, this effect was attenuated by USP42 knockdown (Figure 5F). Regarding total SOD activity in the liver tissue, FGF2 further enhanced CCL4‐induced compensatory SOD activity, whereas USP42 knockdown markedly inhibited this enhancement (Figure 5G). Molecular marker analysis revealed that FGF2 increased the expression of PPARγ target genes including *Sod3*, *Gpx3*, and *Gpx4* (Figure 5H), which was inhibited by USP42 knockdown. These data suggest that USP42 knockdown impedes the anti‐oxidative damaging effect of FGF2 on liver tissues.

To verify these findings, Hepa1‐6 cells were transfected with siNC or siUSP42 after CCL4 treatment. Detection of total ROS levels indicated that FGF2 could prevent ROS bursting. However, siUSP42 transfection and GW9662 administration blocked the ROS‐inhibitory effect of FGF2 (Figure S9A, Supporting Information). The expression of *Sod3* and *Gpx*3 was significantly inhibited by the same treatment (Figure S9B, Supporting Information). On the other hand, the expression of regeneration marker PCNA was promoted by FGF2. However, USP42 knockdown decreased FGF2 induced the PCNA expression (Figure 5I,J). This phenomenon closely resembles what was observed in the cell model, indicating that USP42 functions as a downstream key regulator of FGF2‐mediated liver repair.

### 2.7 Design, Preparation, and Characterization of EV@FGF2‐RSG

**Figure 6:**
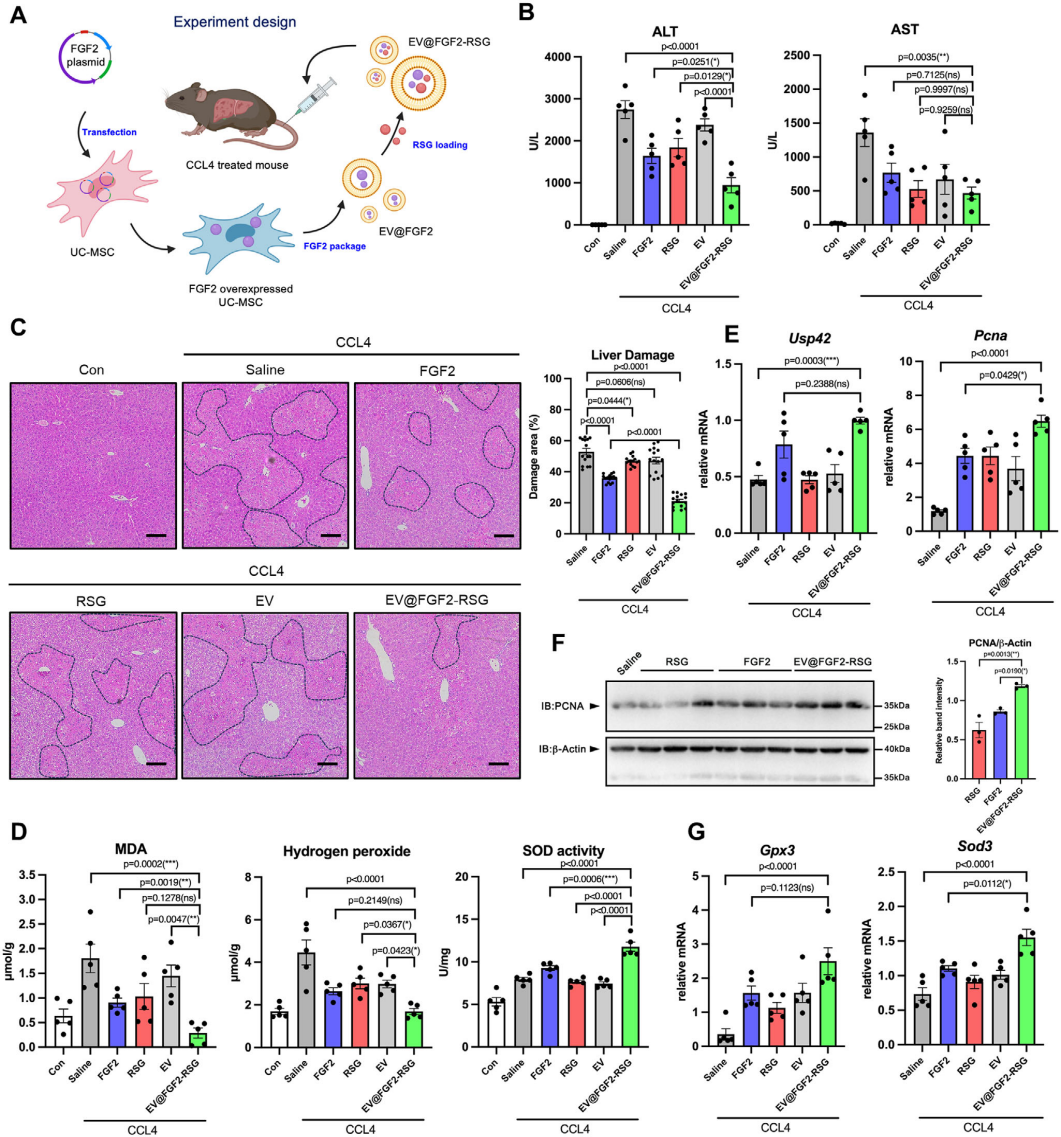
EV@FGF2‐RSG protects the liver from injury and promotes liver regeneration. A) Schematic of the experiment. UC‐MSCs were transfected to the mouse FGF2 coding plasmid, then the UC‐MSC‐secreted EVs containing FGF2 were isolated and the RSG was loaded in EVs to construct the EV@FGF2‐RSG. The EV@FGF2‐RSG (100 µg/per mouse) or the control empty EV (100 µg/per mouse) were injected into mice via the tail vein after 2 h CCL4 treatment. The RSG (5 mg kg^−1^) and FGF2 (0.5 mg kg^−1^) were set as the positive control. B) The activity of ALT and AST was measured by using the fully automated dry biochemical analyzer NX700i (FUJIFILM) (*n* = 5). C) H&E staining of liver tissues (*n* = 5). 100 × magnification, scale bar, 100 µm. Each H&E slide is captured in three fields of view, and the liver injury area in each field of view is calculated to determine the average injury area. Every point in the statistical chart represents the injury area percentage in each field of view. D) The MDA content, the hydrogen peroxides the SOD activity in the liver tissue was measured using the commercial kits (*n* = 5). E) Q‐PCR analysis of the mRNA levels of USP42 and PCNA in liver tissues (*n* = 5). F) Western blotting analysis of PCNA protein levels in liver tissues (*n* = 5). Every lane contains total protein from one or two mice. Every point in the statistical chart represents the band signal intensity of one lane. G) Q‐PCR analysis of the mRNA levels of GPX3 and SOD3 in liver tissues (*n* = 5). Data were analyzed by one‐way ANOVA followed by Tukey's test (B‐G). Data are presented as mean ± SEM. ^＊^ *p* < 0.05. ^＊＊^ *p* < 0.01, ^＊＊＊^ *p* < 0.001.

Given the pivotal role of the USP42‐PPARγ axis in regulating liver regeneration, we have begun considering methods to target this molecular interplay as a basis for developing a novel therapeutic strategy. To activate the USP42‐PPARγ axis, it is essential to first induce the expression of USP42. In our above findings, we have verified that FGF2 is able to initiate the USP42 expression. The second step was to enhance the interaction between USP42 and PPARγ. Unlike other USP proteins, the structure of USP42 has not been thoroughly elucidated, and a selective small‐molecule activator of USP42 has yet to be developed. Therefore, we speculated that the PPARγ ligand possibly possesses the ability to enhance the interaction of USP42 and PPARγ. To test this hypothesis, we overexpressed USP42 and PPARγ in 293T cells and treated the cells with rosiglitazone (RSG), a PPARγ agonist. RSG addition resulted in stronger binding of USP42 to PPARγ, indicating that the full agonist treatment was able to target and activate the USP42‐PPARγ axis (Figure S10A, Supporting Information). The combined use of FGF2 and RSG further augmented this interaction (Figure S10B, Supporting Information). Based on these findings, we designed a nanoparticle that utilized the extracellular vehicles (EVs) to package FGF2 and RSG (EV@FGF2‐RSG) (**Figure** **6**A). By applying this nanoparticle, the FGF2 and RSG could be delivered to liver tissue and activate the USP42‐PPARγ axis.

To develop EV@FGF2‐RSG, umbilical cord‐derived mesenchymal stem cells (UC‐MSC) were transfected with a plasmid coding for murine FGF2. After FGF2 overexpressed in UC‐MSC, EVs including exosomes and microvesicles, were isolated from the culture supernatants using a centrifugation method. To load RSG, the isolated EVs were incubated with RSG at 37 °C for 1 h and the unloaded free compounds were removed by ultrafiltration. Next, we performed characterization of EV@FGF2‐RSG. Nanoparticle tracking analysis (NTA) revealed that these EVs exhibited diameters ranging from 50 to 300 nm, with a mean diameter of ≈177 nm (Figure S10C, Supporting Information). The average zeta potential of EV was −14.3 ± 0.99 mV (Figure S10D, Supporting Information). Transmission electron microscope (TEM) data showed that both empty EV and EV@FGF2‐RSG contained round‐like and cup‐shaped vesicles of 50–150 nm in size with clear membrane structures (Figure S10E, Supporting Information). To further verify that FGF2 was successfully packaged in EV@FGF2‐RSG, western blotting was used to analyze the FGF2 protein level. The results showed that both EV and EV@FGF2‐RSG expressed exosome markers (HSP70, Flotillin‐1, and CD9),^[^ ^31^ ^]^ whereas the FGF2 protein level in EV@FGF2‐RSG was notably higher than that in the control EV (Figure S10F, Supporting Information). Then, to confirm RSG was encapsulated in the EV, we conducted high‐performance liquid chromatography (HPLC) to determine the content of RSG. As Figure S10G shows, both EV@RSG and EV@FGF2‐RSG contained the characteristic peak of RSG. The calculated RSG incorporation rate of EV@RSG and EV@FGF2 was 0.61% ± 0.060% (0.61  ±  0.06 µg RSG/100 µg EV) and 0.60% ± 0.005% (0.6   ±  0.005 µg RSG/100 µg EV), respectively. Moreover, both EV@RSG and EV@FGF2‐RSG could significantly induce the transcriptional activation of PPARγ (Figure S10H, Supporting Information). These data indicated the RSG was successfully loaded into EV@FGF2‐RSG.

Next, we determined the stability of EV@FGF2‐RSG. By storing freshly prepared vesicles (EV@FGF2 and EV@FGF2‐RSG) at −80, −20, and 4 °C, and sampling at different time points for analysis, we used Western blotting to detect EV biomarkers (HSP70 and CD9). We found that vesicles stored at −80 and ‐20 °C showed no noticeable degradation even after 28 days, whereas those stored at 4 °C remained stable for up to 14 days (Figure S10I, Supporting Information). These data demonstrated that both types of vesicles exhibit high stability. In addition, to confirm that the EV protected FGF2 from degradation, we stored the two types of EVs at −80 and 37 °C, and the samples were taken at different time points, dissolved in buffer, and analyzed for released FGF2 content using ELISA. The results showed that EVs stored at −80 °C continuously protected FGF2, with no significant changes in the internal FGF2 content. In contrast, EVs stored at 37 °C showed a decrease in FGF2 content as the storage duration increased (Figure S10J, Supporting Information). Furthermore, we sampled EV@FGF2‐RSG vesicles stored at different temperature conditions and performed a luciferase reporter gene assay. The result showed that EV@FGF2‐RSG stored at −80 °C could continuously activate PPARγ transcriptional activity, indicating that its internal RSG was effectively stored and preserved (Figure S10K, Supporting Information). Together, these experiments demonstrated that EV@FGF2‐RSG exhibits high stability, enabling it to effectively deliver FGF2 and RSG, thereby overcoming barriers in translational medicine. Finally, to verify whether EV@FGF2‐RSG could be uptake by hepatocytes, we used 3,3′‐dioctadecyloxacarbocyanine perchlorate (DIO) to label the empty EV and EV@FGF2‐RSG, and added them into cultured primary hepatocytes in vitro. After 0, 1, 2, 4, and 8 h of incubation, the cells were washed three times to remove the free EVs, and then subjected to flow cytometry to analyze the fluorescence signal. The results showed that 1 h incubation was sufficient for hepatocyte internalization (Figure S10L, Supporting Information). Collectively, these data indicated that EV@FGF2‐RSG is successfully constructed and this nanoparticle could target hepatocytes.

### 2.8 EV@FGF2‐RSG Targets Liver Tissue and Promotes the Regeneration

To assess the liver‐targeting efficacy of EV@FGF2‐RSG, we employed the CCL4‐induced liver injury model as a proof of concept (POC). Two hours after treatment with CCL4 or Vehicle, EV@FGF2‐RSG was intravenously injected into mice. Parallelly, an empty EV was used as the control. First, the biodistribution of EV@FGF2‐RSG and empty EVs was investigated by labeling the EVs with 1,1‐dioctadecyl‐3,3,3,3‐tetramethylindotricarbocyaine iodide (DIR). In both Vehicle and CCL4‐treated mice, the DIR fluorescence was primarily distributed in the liver. Interestingly, compared to the Vehicle group, the liver DIR fluorescence was significantly increased in the CCL4 group, and EV@FGF2‐RSG exhibited a more powerful targeting efficiency than empty EV (Figure S11A–C, Supporting Information). Moreover, it was found that robust amounts of EV@FGF2‐RSG localized to the hepatocytes, and this effect was more pronounced in mice treated with CCL4. (Figure S11D, Supporting Information). These data suggested that EV@FGF2‐RSG preferentially targeted the injured liver.

Next, we evaluated the pro‐regeneration effect of EV@FGF2‐RSG. Systemic administration of FGF2 or RSG, or intravenous treatment with empty EVs were simultaneously conducted as comparable controls. Serum ALT and AST activity assays indicated that EV@FGF2‐RSG exhibited the greatest amelioration of liver injury (Figure 6B). To fully assess the efficacy of EV@FGF2‐RSG, the liver sections were subjected to H&E staining. Quantification of the liver damage area revealed that EV@FGF2‐RSG markedly improved liver damage, and the therapeutic effect was the best among all groups (Figure 6C). Moreover, redox biological marker analysis revealed that EV@FGF2‐RSG notably reduced MDA and hydrogen peroxide levels and enhanced total SOD activity in the liver tissues (Figure 6D). As expected, EV@FGF2‐RSG elevated the mRNA level of USP42, subsequently increasing the gene expression of *Pcna*, *Gpx3*, and *Sod3* (Figure 6E,G). Furthermore, to evaluate the pro‐regenerative efficiency, PCNA protein levels in the liver tissue were measured. As depicted in Figure 6F and Figure S12A (Supporting Information), EV@FGF2‐RSG significantly increased liver PCNA protein levels and number of PCNA‐positive cells. Compared with the systemic administration of FGF2 or RSG, the EV@FGF2‐RSG group showed the highest PCNA expression. We also analyzed the PPARγ protein level, and the results showed that EV@FGF2‐RSG was able to significantly increase the PPARγ protein content in liver tissues (Figure S12B, Supporting Information). To more solid to determine RSG enhanced the efficacy of FGF2 on PPARγ increase and liver regeneration, we included the EV@FGF2 group as the additional investigation. As shown in Figure S12D (Supporting Information), EV@FGF2, like FGF2, can effectively mitigate liver damage. When combined with RSG to form EV@FGF2‐RSG, it demonstrates even greater efficacy in promoting liver repair. The results of MDA content measurement and Q‐PCR analysis of pro‐repair and antioxidant genes further support this conclusion (Figure S12E,F, Supporting Information).

Finally, considering the complex processes involved in liver regeneration, we performed the RNA‐Seq to explore the potential mechanisms of action of EV@FGF2‐RSG in liver repair. The Saline, EV, and EV@FGF2‐RSG were injected into mice after CCL4 treatment, and the liver tissues were subjected to RNA‐seq. Clustering analysis of the whole‐genome expression profiles of the livers revealed that each treatment enriched a unique set of genes (Figure S13A, Supporting Information). USP42 and PPARγ gene expression detection revealed that only EV@FGF2‐RSG could effectively promote USP42 expression, while empty EVs lacked this function (Figure S13B, Supporting Information). This was consistent with our previous findings. Next, we performed pairwise comparisons of saline, EV, and EV@FGF2‐RSG, and conducted gene ontology enrichment (GO) clustering analysis of the differentially enriched gene pathways. By comparing EV and Saline, we found that EV treatment significantly upregulated the cell proliferation‐related pathways, such as cell division and cell cycle. Meanwhile, EV regulates lipid metabolism, including steroid metabolic pathways. On this basis, the comparison between EV@FGF2‐RSG and EV revealed that EV@FGF2‐RSG not only further upregulated pathways related to cell proliferation but also triggered new pathways associated with tissue regeneration and repair, such as anti‐apoptosis and angiogenesis (Figure S13C, Supporting Information). Considering the importance of cell proliferation and anti‐apoptosis pathways in liver tissue regeneration, we detailed and analyzed the expression of all genes within these pathways. As shown in the heatmaps of Figure S13D (Supporting Information), EV@FGF2‐RSG further enhanced the expression of cell proliferation genes on top of the proliferative effects induced by EV. Additionally, EV@FGF2‐RSG induced the expression of several key anti‐apoptotic genes, such as *Ppard* and *Kif14*. These results collectively indicate that EV@FGF2‐RSG promotes liver self‐repair by effectively enhancing hepatocyte proliferation and protecting hepatocytes from cell death. In the anti‐apoptotic pathway, there are several genes related to redox balance. As shown in Figure S13E (Supporting Information), EV@FGF2‐RSG significantly upregulated the expression of most antioxidant genes, such as *Loxl2*, *Gpx3, Sod3*, and *MMP9/MMP13*, indicating that EV@FGF2‐RSG enhanced the antioxidant capacity of the liver. To further explore the potential mechanisms, we conducted Kyoto Encyclopedia of Genes and Genomes (KEGG) and Gene Set Enrichment Analysis (GSEA) and found that EV@FGF2‐RSG was able to regulate pathways such as Ras, MAPK, and PI3K, as well as pathways involved in lipid and toxic substance metabolism (Figure S13F,G, Supporting Information). These findings indicated that EV@FGF2‐RSG promoted liver repair through multifunctional mechanisms, and regulated various physiological functions to facilitate liver regeneration. Together, these data indicated that EV@FGF2‐RSG had therapeutic effects against liver injury.

## 3 Discussion

The regenerative capacity of the liver is vital for maintaining metabolic homeostasis and systemic health, particularly during acute toxic insult or chronic conditions. Understanding the mechanisms governing liver repair has significant potential in the context of aging, metabolic diseases, and critical illnesses. For instance, in critical illness, acute liver failure (ALF) and drug‐induced liver injury (DILI) represent life‐threatening conditions where timely liver repair determines outcomes. Studies have revealed that the mortality rates of ALF remain above 80%, emphasizing the need for therapeutic strategies to enhance repair.^[^ ^32^ ^]^ Besides, in aging and metabolic diseases, liver function declines due to reduced regenerative capacity and increased susceptibility to damage, contributing to systemic metabolic dysregulation. One study found that in aging populations, the risk of liver fibrosis progression increases due to reduced regenerative capacity and chronic low‐grade inflammation, as observed in studies on non‐alcoholic fatty liver disease (NAFLD) and other metabolic liver disorders.^[^ ^33^ ^]^ Notably, ≈12.5% of NAFLD patients experience incident non‐fibrotic non‐alcoholic steatohepatitis (NASH), with disease progression associated with a significantly higher risk of developing end‐stage liver disease (ESLD).^[^ ^34^ ^]^ Regenerative dysfunction exacerbates the progression of NASH and cirrhosis in patients with NAFLD. Enhancing the liver repair pathways can mitigate age‐related fibrosis and maintain metabolic health. Therefore, investigating liver repair mechanisms offers a promising avenue for addressing age‐associated decline, metabolic disease burden, and critical illnesses.

The post‐translational modifications (PTMs) of PPARγ endow this nuclear receptor with diverse and complex biological functions in regulating metabolism and inflammatory response.^[^ ^35^ ^]^ Previous studies have demonstrated that Ser273 phosphorylation selectively determines the insulin sensitivity gene expressions in adipocytes.^[^ ^36^ ^]^ The Thr166 phosphorylation and Lys268/293 acetylation control the beige adipocyte differentiation.^[^ ^13^, ^35^ ^]^ The Lys364 SUMOylation regulates the PPARγ and co‐factors interactions, which influences the macrophage inflammatory gene expression.^[^ ^35b^ ^]^ Additionally, the ubiquitination of PPARγ has been shown to impact protein stability and its related metabolic features in adipocytes and cancer cells.^[^ ^37^ ^]^ To date, few PPARγ PTM functions have been reported in liver tissue. In the present study, we discovered that PPARγ T166A (Threonine 166 mutated to alanine, TA) mice exhibited enhanced liver regeneration capacity, and this phenomenon was tightly related to TA protein stability. Thus, it is highly likely that ubiquitination modification is involved in this molecular process. From this standpoint, we compared the interactive protein profiles between WT and TA, which led us to find the USP42 interplayed with TA. Subsequent investigations proved that USP42 was a deubiquitinating enzyme in the primary hepatocyte and hepatocyte cell line. These findings not only elucidate the function of PPARγ ubiquitination in liver tissue but also hints that T166 phosphorylation might be a trigger to regulate ubiquitination. In fact, multiple PTMs in PPARγ or other transcriptional factors can coordinate with intimate interdependencies, and then precisely modulate their diverse properties. For instance, PPARγ Lys268/Lys293 acetylation and Ser273 phosphorylation are interdependent.^[^ ^35^, ^38^ ^]^ Ser315/Ser362 phosphorylation in p53 assists ubiquitination, which induces p53 degradation.^[^ ^39^ ^]^ Future studies should focus on the exact mechanism of T166 phosphorylation in regulating PPARγ ubiquitination, and identify other ubiquitination regulatory proteins besides USP42 in hepatocytes.

USP42 has been less studied than other DUBs. In 2006, a case report first uncovered USP42 and RUNX1 fusion protein in acute myeloid leukemia (AML), and confirmed that this DUB was expressed in bone marrow and some cancer cells.^[^ ^40^ ^]^ Over the next two decades, the biological functions of USP42 in several cell types have partially been elucidated. In embryonic development, USP42 has been showed to be expressed in embryonic stem cell and participates in embryogenesis and spermatogenesis.^[^ ^41^ ^]^ In microglial cells, USP42 stabilizes cellular TRIM21 for anti‐viral response upon the Japanese Encephalitis Virus (JEV) infection.^[^ ^42^ ^]^ Tumor cell regulatory functions have also been reported in gastric cancer, colon cancer, and osteosarcoma cells, and the molecular mechanisms involved in stabilizing WNT signaling, p53 transcription, and nuclear speckle mRNA splicing.^[^ ^27^, ^43^ ^]^ To the best of our knowledge, this study is the first to report the role of USP42 in liver damage and regeneration. USP42 is highly expressed in hepatocytes, and the USP42 expression is highly correlated with liver regeneration in mouse and human samples. USP42 knockdown markedly delays regeneration and aggravates ROS‐induced liver injury. USP42 promotes PPARγ stability and drives the transcription of proliferative and anti‐ROS genes, which enhances liver repair. These new findings not only confirm the critical role of USP42 in liver function but also link USP42 to PPARγ at the molecular level. In addition to its functions in metabolic regulation, PPARγ also plays a crucial role in redox balance and cell proliferation.^[^ ^29^, ^44^ ^]^ Under certain conditions, the cellular PPARγ senses extracellular signals and activates antioxidant enzyme and mitogen expression. However, the mechanism by which PPARγ senses extracellular signals is not yet well defined. In our model, when the liver started to repair, USP42 mRNA and protein levels were both significantly increased, suggesting that USP42 acts as a sensor that links the regeneration signal to PPARγ activation. Moreover, our results are similar to those observed in some cancer cells, in which USP42 overexpression promoted PCNA and cyclin gene transcription.^[^ ^45^ ^]^ Regeneration and tumorigenesis share common biological processes, including DNA repair, cell cycle initiation, and resistance to ROS damage.^[^ ^46^ ^]^ Thus, our findings may be applicable to liver cancer.

While the attractiveness of using FGF2 and TZD as therapeutic is evident, there are still several limitations. First, as a protein drug, FGF2 is susceptible to chemical and biological instability under intravenous administration.^[^ ^52^ ^]^ Second, systemic treatment with FGF2 may induce abnormal ectopic cell proliferation in tissues beyond the liver.^[^ ^53^ ^]^ Third, systemic TZD treatment possibly causes side effects, such as bone rarefaction, and heart failure.^[^ ^14b^ ^]^ In our CCL4‐induced liver injury model, it was unlikely that acute treatment with FGF2 or TZD caused serious adverse effects. However, combination therapy regimens are limited to the treatment of chronic liver damage. Therefore, it is essential to construct a delivery system that improves the efficiency of FGF2 and TZD targeting. EVs derived from mesenchymal stem cells (MSC) are ideal nanocarriers for drug delivery.^[^ ^54^ ^]^ Recent studies have demonstrated that EVs from MSC exhibit various therapeutic actions on tissue regeneration and inflammatory regulation.^[^ ^55^ ^]^ Moreover, compared to existing nano‐delivery carriers, MSC‐derived EVs have several advantages, such as higher safety, lower immunogenicity, and the ability to cross biological barriers.^[^ ^56^ ^]^ Here, to protect FGF2 from degradation in blood and targeted delivery of FGF2/RSG to the liver, we constructed a UC‐MSC‐based platform to generate EV@FGF2‐RSG. In our study, FGF2 and RSG were efficiently packaged into EVs, and EV@FGF2‐RSG effectively targeted the liver, particularly injured liver tissues. This phenomenon may be due to the upregulation of integrins and adhesion molecules in damaged tissues, which enables biological vesicles to adhere effectively to injured tissues.^[^ ^31b^ ^]^ It is noteworthy that the pharmacological efficacy of EV@FGF2‐RSG on ROS inhibition and liver regeneration is significantly higher than those observed in systemic FGF2 or RSG treatment, which suggests that EV@FGF2‐RSG is a potential nanodrug for liver repair. The ongoing work should test EV@FGF2‐RSG on chronic liver disease, further enhancing its translational medical application value.

## 4 Experimental Section

### Animal Experiments

The male C57BL/6J mice (18–20 g) were purchased from the GemPharmatech Co., Ltd. The TA mice were bred and genotyped as previously described.^[^ ^13^ ^]^ Mice were housed in the experimental animal room of Wenzhou Medical University in a controlled environment (23 ± 2 °C, 50%–60% humidity, 12 h light/dark cycle) under specific pathogen‐free (SPF) conditions. For the liver injury model, the mice were intraperitoneally injected with 10% CCL4 (10 006 464, Sinopharm, China) (10 mL kg^−1^, dissolved in olive oil). In exosome‐related animal experiments, the mice were first treated with CCL4, after 2 h administration, the mice were intravenously injected with FGF2 (0.5 mg kg^−1^), RSG (5 mg kg^−1^), EV (100 µg/per mouse), EV@FGF2 (100 µg/per mouse) and EV@FGF2‐RSG (100 µg/per mouse) via the tail vein once. The dosage of exosomes (100 µg per mouse) refers to the amount determined by BCA quantification of the extracted exosomes, calculated based on the total protein content of the EVs. Each mouse was injected with 100 µg of EVs.The total protein concentration of EV or EV@FGF2‐RSG was quantified with a BCA assay kit (P0011, Beyotime, China). After treatment, all mice were anesthetized and the plasma was collected for biochemical analysis. The liver tissues were collected for histopathological, quantitative real‐time PCR (q‐PCR), and Western blotting analysis. Animal welfare and experimental procedures were carried out in strict compliance with the “Guide for the Care and Use of Laboratory Animals” (Ministry of Science and Technology of China) and the related ethical regulations of Wenzhou Medical University. Ethical approval was obtained under the accreditation number (Approval Number: wydw2022‐0714).

### Cell Culture

HEK293T, Hepa1‐6, and AML‐12 were purchased from the Stem Cell Bank (Chinese Academy of Sciences, China), and cultured in Dulbecco's Modified Eagle's Medium (DMEM) supplemented with 10% fetal bovine serum (BC‐SE‐FBS08, Sbjbio, China), 100 U mL^−1^ penicillin and 100 µg mL^−1^ streptomycin (60162ES76, Yesen, China). Cells were cultured in a humidified atmosphere with 5% CO2 at 37 °C. For primary hepatocyte isolation and culture, the anesthetized mice were perfused with perfusion medium (5 mM HEPES, 120 mM NaCl, 4.8 mM KCl, 23.8 mM NaHCO_3_, 1.2 mM MgSO_4_, 1.2 mM KH_2_PO_4_, 0.1% Glucose and 1 mM EGTA), followed by liver digest medium (0.05% collagenase Type IV, 5 mM HEPES, 120 mM NaCl, 4.8 mM KCl, 23.8 mM NaHCO_3_, 1.2 mM MgSO_4_, 1.2 mM KH_2_PO_4_, 0.1% Glucose and 5 mM CaCl_2_) through the portal vein. Then, the livers were excised and minced, and the tissues were filtered with a 100 µm cell strainer. Primary hepatocytes were next centrifuged at 50 g for 5 min and cultured in William'E medium (Gibco, 12 551 032) supplemented with 10% FBS, 1 mM of GlutaMAX (Gibco, 35 050 061), 100 U mL^−1^ penicillin and 100 µg mL^−1^ streptomycin for 4 h. For cell experiments, the primary hepatocytes were maintained in William'E medium containing 10% BSA, 100 U mL^−1^ penicillin, and 100 µg mL^−1^ streptomycin. UC‐MSC were cultured in alpha‐MEM medium supplemented with 10% fetal bovine serum.

### Chemicals and Antibodies

The following chemicals were used in the present study: MG‐132 (MedChemExpress, HY‐13259), Cycloheximide (CHX, MedChemExpress, HY‐12320), Cumene hydroperoxide (CuOOH, MACKLIN, C805078), GW9662 (MedChemExpress, HY‐16578), rosiglitazone (MedChemExpress, HY‐17386), EdU (Beyotime, C0078S), CFSE (ThermoFisher, 65‐0850‐84), DiR (YEASEN, 40757ES25), DiL (YEASEN, 40718ES60), DiO (YEASEN,40725ES25). Recombinant mouse FGF2 protein (Z03016) was purchased from Genscript Co., Ltd.

The following commercial antibodies were used in the present study: anti‐PPARγ rabbit mAb (#2443, Cell Signaling Technology), anti‐PPARγ mouse mAb (sc‐7273, Santa Cruz), anti‐USP42 Rabbit pAb (A15911, ABclonal), anti‐Ubiquitin Rabbit mAb (#43 124, Cell Signaling Technology), anti‐K63‐linkage Specific Polyubiquitin Rabbit pAb (A18164, ABclonal), anti‐K48‐linkage Specific Ubiquitin Rabbit mAb (A3606, ABclonal), anti‐PCNA mouse mAb (sc‐56, Santa Cruz), anti‐FGF2 rabbit pAb (11234‐1‐AP, Proteintech), anti‐HSP70 rabbit pAb (#4872, Cell Signaling Technology), anti‐Flotillin‐1 rabbit pAb (#3253, Cell Signaling Technology), anti‐CD9 rabbit mAb (#13 174, Cell Signaling Technology), anti‐β‐actin (700 068, Zenbio). The anti‐FGF2 neutralizing antibody was purchased from Merck (Sigma‐Aldrich) (05‐117).

### Malondialdehyde (MDA), Glutathione (GSH), Superoxide Dismutase (SOD), and Hydrogen peroxide Assays

The assay kits of malondialdehyde (MDA) (S0131S), total superoxide dismutase (SOD) activity (S0101S), and hydrogen peroxide (S0038) were purchased from Beyotime Biotechnology. Alanine aminotransferase (ALT) assay kit (C009‐2‐1), Aspartate aminotransferase (AST) assay kit (C010‐2‐1), and glutathione (GSH) (A006‐2‐1) kit were purchased from Nanjing Jiancheng Bioengineering Institute (Nanjing, China). The procedure was performed according to the manufacturer's instructions.

### Fluorescence Staining and Confocal Microscopy Analysis

For staining TUNEL or PCNA, the liver tissues were first fixed in 10% formaldehyde for at least 24  h and embedded in paraffin, and the sections (5‐µm) were cut and stained with anti‐PCNA antibody or TUNEL kit (G3250, Promega). For staining lipid droplets, the liver tissues were subjected to the frozen section, and the lipid droplets were labeled with 2  µM BODIPY 493/503 (ThermoFisher, D3922) for 15  min. For staining USP42 and PPARγ, the frozen sections of liver tissues were applied to the anti‐USP42 and anti‐PPARγ antibody staining. Images were visualized and photographed using a Nikon A1R confocal laser scanning microscope.

### Mass Spectrometry Analysis

AML‐12 cells were plated in 10‐cm dishes and allowed to adhere overnight prior to transfection with the Lentivirus containing WT or TA PPARγ plasmids. The Plenti 6/V5 plasmids which encoded the WT or TA PPARγ genes were packaged into lentivirus as described previously.^[^ ^57^ ^]^ Briefly, psPAX2, pMD2.G and pLenti 6/V5 plasmids were transfected into HEK293T cells. After 48 h transfection, the cell culture supernatant was collected, and the supernatant was a concentration reagent (ExCell, EMB810A‐1). The concentrated lentivirus was added into AML‐12 cells with supplementary with 10 µg mL^−1^ polybrene (Yeasen, 40804ES76). After 48 h transfection, the AML‐12 cells were lysed with IP lysis buffer (Cell Signaling Technology, 9803S). Total protein (1 mg) was pre‐cleared with 1 µg mouse IgG and 20 µL Protein A+G agarose (ab193262, Abcam, UK) for 1 h at 4 °C. Then, the PPARγ protein was enriched by using an anti‐PPARγ antibody (Santa Cruz, sc‐7273) with Co‐IP. The enriched protein complex in protein A+G agarose was next obtained by adding the elution buffer (glycine solution, pH 2.5) followed by neutralization with 1M Tris solution (pH 8.0).

Total eluates (80µL) were incubated with 160 µL reduction‐alkylation buffer [50mM Tris‐HCl, pH8.0, 2M Urea, 1 mM Dithiothreitol (DTT), 3mM iodoacetamide (IAA)] in the dark at room temperature for 1 h. After quenching IAA with 3 mM DTT, trypsin (Promega) was added to samples at a 1:100 enzyme: substrate ratio and incubated overnight at 37 °C. Peptides were treated with 1% formic acid and desalted. The resulting peptides were concentrated on ZipTip C18 pipette tips (Millipore) and eluted in a final 20 uL solution of 0.1% formic acid prior to mass spectrometry. For MS/MS analysis, digested peptides were analyzed by LC‐MS/MS on a Thermo Scientific Velos Pro ion trap MS system equipped with a Proxeon Easy nLC II high‐pressure liquid chromatography and autosampler system. The system delivered a gradient from 5% to 30% ACN in 0.1% formic acid over 1 h and collected data in a data‐dependent fashion. Raw MS files were analyzed by Protein Prospector (http://prospector.ucsf.edu/), version 5.10.1. The searched data was then scored for protein‐protein interactions (PPIs) using the MiST algorithm.^[^ ^58^ ^]^

### RNA Extraction and Quantitative Real‐Time PCR (Q‐PCR)

Total RNA was isolated from liver tissues or culture cells using TRIzol reagent (R401‐01, Vazyme, China), and cDNA was synthesized using a Hiscript III Reverse Transcriptase (R302‐01, Vazyme, China) kit in accordance with the manufacturer's instructions. Q‐qPCR was performed on a QuantStudio 7 Flex (Applied Biosystems) using AceQ qPCR SYBR Green Master Mix (Vazyme, Q111‐02). The expression of the measured genes was normalized to the β‐Actin or GAPDH mRNA expression level. For ChIP‐QPCR, the cells were applied to the ChIP assay by using the SimpleChIP Enzymatic Chromatin IP Kit (Cell Signaling Technology, #9003) with the manufacturer's instructions. The sequences of the primers used for Q‐PCR are shown in Table S2 (Supplementary Table2).

### RNA Interference

Hepa1‐6 cells and primary hepatocytes were transfected with siUSP42 or siNC scrambled (negative) control (designed and synthesized by HanBio Co., Ltd) by using INTERFERin (Polyplus, 101 000 028) for 48 h. All the primers for siRNA sequences used in this study are shown in Table S3 (Supplementary Table3).

### Histological Analyses

Liver tissues were fixed overnight in 4% formaldehyde (G1101, Servicebio, China) and embedded in paraffin. Then, the tissues were cut into 5µm‐thick slices and stained with hematoxylin and eosin (H&E). After dewaxing, hydration, dyeing, dehydration, transparency, and sealing, samples were photographed using Panoramic Scan (3DHISTECH, Hungary). The liver damage area was analyzed using the Image J software.

### Western Blotting, Co‐Immunoprecipitation (Co‐IP) and Ubiquitination Assays

Proteins were extracted using RIPA lysis buffer (P0013B, Beyotime, China) containing protease inhibitor cocktail (HY‐K0010, MCE, USA) and phosphatase inhibitor cocktail II (HY‐K0022, MCE, USA). After centrifugation at 4 °C, the protein concentration was determined using a BCA assay kit (P0011, Beyotime, China). The extracted proteins were separated by electrophoresis on 8%, 10%, or 12% SDS‐PAGE gels and then transferred to polyvinylidene difluoride membranes (PVDF) (PB9220, Invitrogen, USA) for immunoblotting. The primary antibodies used were mentioned above. The secondary antibodies was HRP‐labeled goat anti‐rabbit IgG (A0208, Beyotime, China) or HRP‐labeled goat anti‐mouse IgG (A0216, Beyotime, China).

For Co‐IP, the cells were lysed in IP lysis buffer (Cell Signaling Technology, 9803S). Total protein (500 µg mg^−1^) was pre‐cleared with 1 µg rabbit/mouse IgG and 20 µL Protein A+G agarose (ab193262, Abcam, UK) for 1 h at 4 °C. Total protein was isolated by centrifugation and 1 µg anti‐USP42 antibody (A15911, ABclonal) was added. Following overnight incubation at 4 °C, 30 µL Protein A+G agarose was used to purify the immune complex, following which the agarose was washed 5 times in wash buffer (150 mM NaCl, 1 mM EDTA, 20 mM Tris, 1% Triton X‐100, pH 8.0). Protein was eluted in 1× SDS‐PAGE loading buffer at 100 °C for 10 min.

For ubiquitination assay, the cultured cells were treated with 10 µM MG‐132 for 12 h. The cells were lysed in IP lysis buffer (Cell Signaling Technology, 9803S) and the total protein (1 mg) was pre‐cleared with 1 µg mouse IgG and 20 µL Protein A+G agarose (ab193262, Abcam, UK) for 1 h at 4 °C. Then, 1 µg anti‐PPARγ antibody (sc‐7273, Santa Cruz) was added. Following overnight incubation at 4 °C, 30 µL Protein A+G agarose was used to purify the immune complex, following which the agarose was washed 5 times in wash buffer (150 mM NaCl, 1 mM EDTA, 20 mM Tris, 1% Triton X‐100, pH 8.0). Protein was eluted in 1× SDS‐PAGE loading buffer at 100 °C for 10 min and applied to Western blotting analysis.

In the initial experiment (Figure S7D, Supporting Information), we tested 50 and 100 ng mL^−1^ FGF2 to determine the minimal effective concentration for increasing PPARγ protein levels. Based on these results, we used 100 ng mL^−1^ as the baseline concentration for subsequent experiments (Figure S7E–G, Supporting Information) and further explored the effects of a higher concentration (200 ng mL^−1^) to assess the dose‐response relationship.

### Plasmids, Lentiviruses, and Adeno‐Associated Virus 8

pLenti 6/V5 was used to ectopic express the USP42, PPARγ and FGF2. This plasmid was also used to generate the lentivirus carry WT and TA PPARγ. pLenti 6/V5 plasmid was purchased from Invitrogen (Catalog nos. K4955‐00). The sequences of mouse USP42, PPARγ, and FGF2 were obtained from the NCBI database, with their respective Gene IDs being 76 800 (USP42), 19 016 (PPARγ) and 14 173 (FGF2). Their coding sequences were fully synthesized by GenScript and inserted into the multiple cloning site of the pLenti 6/V5 vector using ClonExpress II One Step Cloning Kit (Vazyme, C112‐01), thereby completing the plasmid construction. The mouse USP42 cDNA (WT or C119A mutant) was cloned into pLenti 6/V5 with a Flag tag. The mouse PPARγ was cloned into pLenti 6/V5 with an HA tag. For lentivirus generation, the pLenti 6/V5 was co‐transfected with psPAX2 and pMD2.G into HEK293T cells. After 48 h transfection, the cell culture supernatant was collected, and the supernatant was concentration reagent (ExCell, EMB810A‐1) to prepare the lentivirus.

AAV8‐shNC and AAV8‐shUSP42 were generated by VectorBuilder Co., Ltd (China). For the generation of AAV8‐shUSP42 mice, C57BL/6 mice were injected with AAV8‐shUSP42 (1 × 10^11^ viral particles/200 µL PBS) via the tail vein. After three weeks for a knockdown, AAV8‐shUSP42 mice were subjected to further study.

### PPARγ Gene−Luciferase Reporter Assay

HEK293T cells were transfected with PPARγ2, PPRE‐Luc, and pRL at a molar ratio of 50:50:1 using a Lipofectamine 2000 kit (11 668 019, Invitrogen, USA). After an 8 h transfection period, the culture medium was replaced with fresh DMEM containing 10% FBS. RSG, EV@RSG, and EV@FGF2‐RSG were added to the culture wells and incubated for 24 h. Cells were collected and lysed in luciferase lysis buffer, and the luciferase activity of the cells was measured using the dual‐luciferase reporter assay system (E1910, Promega, USA) on the Modulus luminescence detector (Turner Biosystems, USA).

### EV@FGF2‐RSG Construction and Characterization

First, the UC‐MSCs were transfected with FGF2 lentivirus. After 48 h transfection, the overexpression of FGF2 was verified by western blotting or Q‐PCR. Then, UC‐MSC‐derived EV@FGF2 was purified from a cell culture supernatant without FBS by ultracentrifugation as previously reported.^[^ ^57^ ^]^ Briefly, FGF2 lentivirus transfected UC‐MSCs were cultured in fresh MEMα medium (L571KJ, BasalMedia, China) without FBS. After 24 h, the supernatant was collected and centrifuged at 300 g and 2000 g for 10 min to remove cells and dead cells, respectively. The supernatant was then centrifuged at 10000 g for 30 min to eliminate cell debris. The final supernatant was then ultracentrifuged at 100 000 g for 70 min to collect the EV@FGF2. For loading RSG, 200 µg of EV@FGF2 (200 µL) was pipetted into an EP tube and 1 mg of RSG was added. Incubation was then performed at 750 rpm, 37 °C for 2 h. After incubation, the mixture was subjected to ultrafiltration by using the 100 kD ultrafiltration tube (UFC510008, Merck, Germany) to remove excess RSG and obtain the EV@FGF2‐RSG. The constructed EV@FGF2‐RSG was applied to characterization or cell/animal experiments.

For the characterization of EV@FGF2‐RSG, the size distribution of this nanoparticle was first detected by NTA (Particle Metrix, Germany). Then, the morphology was examined by TEM (HITACHI, Japan). The Zeta potential of EV@FGF2‐RSG was determined on the Zetasizer Nano (Malvern Panalytical, UK). EV markers (HSP70, Flotillin, and CD9) and encapsulated FGF2 proteins were detected by Western blotting. The RSG loading capacity of EV@FGF2‐RSG was determined using high‐performance liquid chromatography (HPLC) (Agilent, USA). Briefly, EV@FGF2‐RSG was precipitated with three times the volume of HPLC‐grade methanol (106 007, Merck, Germany) to remove the protein. The supernatant was freeze‐dried and reconstituted with 100 µL of methanol. Chromatographic analysis conditions: the detector was a diode array detector (DAD) with a detection wavelength of 245 nm, Absolute AQ‐C18 (4.6 × 150 mm), and a flow rate of 0.9 mL mi^−1^n. The mobile phases were 0.1% formic acid aqueous solution in phase A and acetonitrile in phase B. The gradient elution procedure was as follows: 0–7 min, 50% B; 7–8 min, 50%–95% B; 8–12 min, 95% B. The sample injection volume was set at 1 µL.

### Internalization Assay of EV@FGF2‐RSG In Vitro

Primary hepatocytes were cultured in 12‐well plates and left to grow to 80% for fluid exchange and staining. EV and EV@FGF2‐RSG were labeled with DIO (1:500) (40725ES10, YEASEN, China) and incubated at 37 °C for 1 h. Subsequently, DIO‐labeled EVs were subjected to centrifugation (100 000 g, 90 min) in an ultra‐high‐speed centrifuge to remove free dye. Then, 100µl of labeled EV and EV@FGF2 were added to culture cells, and the internalization efficiency was tested by flow cytometry at different time points (1, 2, 4, and 8 h).

### Distribution of EV@FGF2‐RSG In Vivo

Tissue distribution of EV and EV@FGF2 was performed on normal and C57BL/6J mice with acute liver injury induced by CCL4. EV and EV@FGF2 were labeled using DIR dye (1:500) (40757ES25, YEASEN, China) and incubated at 37° for 1 h. After incubation, the free dye was removed by ultracentrifugation (100 000 g, 4°, 90 min). Each mouse was injected with 100 µg of EV or EV@FGF2‐RSG via the tail vein. In vivo imaging analysis was performed at different time points (24 and 48 h) by using an IVIS imager (PerkinElmer, USA). Then, mice were euthanasia and the organs (heart, liver, spleen, lung, and kidney) were isolated for live imaging analysis.

### Cellular Uptake of EV@FGF2‐RSG In Vivo

DIL‐labeled (40718ES50, YEASEN, China) EV or EV@FGF (100 µg) were injected in normal and C57BL/6J mice with acute liver injury induced by CCL4. After 24 h, liver tissue was collected for confocal microscopic analysis. Immunostaining against albumin (16475‐1‐AP, Proteintech, China) was used to label the hepatocytes. Finally, DIL^+^Albumin^+^ cells were counted using ImageJ (NIH, USA).

### Cell Proliferation Assay

Cell proliferation assays were performed using CCK‐8, CFSE, and EdU methods, respectively. For the CCK‐8 assay, USP42‐OE and USP42‐KD cells were plated in 96‐well plates (2 × 10^4^ cells mL^−1^) in a cell culture incubator at 37 °C. After 12 h of incubation, GW9662 was added to the USP42‐KD cells at a final concentration of 2 µM. After a further incubation of 48 h, 10 µL of CCK‐8 solution (A311‐01, Vazyme, China) was added to the 96‐well plates, and the plates were incubated for 3 h. Finally, the absorbance values at 450 nm were detected using a MicroplateReader (AMR‐100, Allsheng, China).

For CFSE, USP42‐OE and USP42‐KD cells were stained with CFSE dye (65‐0850‐84, ThermoFisher, USA) at a final concentration of 10 µM and incubated at 37 °C for 15 min. The cells were washed with a complete medium to remove unbound CFSE‐free dye. Labeled USP42‐OE and USP42‐KD cells were then plated in 12‐well plates (1 × 10^6^ cells mL^−1^). After 12 h, GW9662 was added to the USP42‐KD cells at a final concentration of 2 µM. The cells were incubated for a further 48 h, followed by fluorescence detection by flow cytometry.

As for the EdU assay, USP42‐OE and USP42‐KD cells were plated in 12‐well plates (1 × 10^6^ cells mL^−1^) in a cell culture incubator at 37 °C. After 12 h of incubation, GW9662 was added to the USP42‐KD cells at a final concentration of 2 µM. After a further incubation of 48 h, the cells were stained with EdU dye (C0078S, Beyotime, China). The following experiments were performed according to the instructions of the EdU kit. Finally, photographs were taken using a fluorescence microscope (BX53, Olympus, Japan).

### Reactive Oxygen Species (ROS) Content Analysis

Cells were washed twice with PBS. The DCF probe (Beyotime, S0033S) was used to evaluate the total ROS level in cells according to the manufacturer's instructions. For analyzing the mitochondrial ROS, the MitoSOX probe (M36008, ThermoFisher, USA) was added at a final concentration of 5 µM and incubated for 30 min at room temperature in the dark. Cells were washed twice with PBS to remove residual probes. Cells were resuspended by adding 200 µL of PBS and detected by flow cytometry.

### cDNA Chip

The cDNA samples of 30 hepatocellular carcinoma patients were purchased from OUTDO Biotech Co., Ltd. The information on the patient samples is shown in Table S4 (Supplementary Table4). Q‐PCR was performed using AceQ qPCR SYBR Green Master Mix (Q111‐02, Vazyme, China) on QuantStudio 7 Flex (Applied Biosystems).

### RNA‐Sequencing and Data Analysis

Liver tissue samples were collected, snap‐frozen in liquid nitrogen, and stored at −80 °C. Total RNA was extracted TRIzol reagent (Invitrogen). Libraries were conducted according to the manufacturer's protocol. The libraries were sequenced on a Novaseq x plus (Illumina) in LC‐Bio Technology Co., Ltd (Hangzhou, China), and the reads were aligned to the GRCm38.97 genome (ENSEMBL) using Hisat2 (v2.2.1). Gene expression levels were calculated as fragments per kilobase of transcript per million mapped reads (FPKM). Differentially expressed genes (DEGs) were identified using DESeq2 (v1.38.0) in R (v4.3.1). Genes with an adjusted *p*‐value < 0.05 and log2 fold chang≥ 1 were considered significantly differentially expressed. Functional enrichment analysis of DEGs was performed using the clusterProfiler (v4.8.0) package in R. GO terms for biological processes, molecular functions, and cellular components were identified with the significance threshold set at adjusted *p*‐value < 0.05. Kyoto Encyclopedia of Genes and Genomes (KEGG) pathway analysis was conducted to explore enriched signaling pathways. Gene Set Enrichment Analysis (GSEA) was conducted using the fgsea (v1.26.0) package in R. Visualization was performed by OmicStudio (v3.6) developed by LC‐Bio Technology Co., Ltd (Hangzhou, China). The raw reads have been deposited in the NCBI Gene Expression Omnibus database with the accession number GSE286997.

### Statistical Analysis

Statistical analysis was performed using the GraphPad Prism 10.0 software. The R version 4.4.1 was used for RNA‐Seq data processing and analysis. Error bars indicate the standard error of the mean (S.E.M.) unless otherwise indicated. The sample size (n) for each statistical analysis is explicitly mentioned for all experiments in the figure legends. The unpaired Student's t‐test was used for two‐group comparisons, and the one‐way ANOVA followed by Tukey's test was used for multiple‐group comparisons. The two‐way ANOVA followed by Bonferroni's test was used for the CCK‐8 assay, PPARγ stabilization assay, and body/liver weight. *P  <  0.05, **P  <  0.01, ***P  <  0.001.

## Conflict of Interest

The authors declare no conflict of interest.

## Author Contributions

N.Y. and Q.T. contributed equally to this work. L.X.K., H.Z.F., S.P.P., and Y.N.F. designed the research and analyzed the data. Y.N.F., T.Q., L.Z.L., W.S.X., C.N., W.Z., J.X.Q., Z.X.Q., and X.W.J. performed the experiments. Y.N.F. and T.Q. performed the bioinformatics analysis and drew the graph abstract. Y.M.Y., Z.L.W., and W.M.Y. performed the histological and western blotting analysis. H.Z.F., L.X.K., S.P.P., N.J.L., and S.W.J. provided scientific suggestions and contributed to the manuscript revision. L.X.K., H.Z.F., and S.P.P. supervised the project. L.X.K., H.Z.F., and Y.N.F. wrote the manuscript. All authors critically reviewed the manuscript and approved the final article.

## Supporting information



Supporting Information

Supplementary Table1

Supplementary Table2

Supplementary Table3

Supplementary Table4

## Data Availability

The data that support the findings of this study are available from the corresponding author upon reasonable request.
