# Supplementary material for: FGF2 Mediated USP42‐PPARγ Axis Activation Ameliorates Liver Oxidative Damage and Promotes Regeneration
Source: Adv Sci (Weinh). 2025 Mar 17;12(18):2408724. doi: 10.1002/advs.202408724 (PMC12079552; doi:10.1002/advs.202408724)
Supplement: Supplementary file 1 — Supporting Information [file ADVS-12-2408724-s004.pdf]

ADVANCED SCIENCE
Open Access

Supporting Information

for *Adv. Sci.*, DOI 10.1002/advs.202408724

FGF2 Mediated USP42-PPARγ Axis Activation Ameliorates Liver Oxidative Damage and Promotes Regeneration

Nanfei Yang, Qiang Tian, Zhenli Lei, Shuxin Wang, Nan Cheng, Zhen Wang, Xianqin Jiang, Xuqun Zheng, Wenjing Xu, Minyan Ye, Longwei Zhao, Meiyun Wen, Jianlou Niu, Weijian Sun, Pingping Shen, Zhifeng Huang* and Xiaokun Li**

Supporting Information

FGF2 mediated USP42-PPARγ axis activation ameliorates liver oxidative damage and promotes regeneration

Authors

Nanfei Yang[1,2,3, †], Qiang Tian[2, †], Zhenli Lei[1], Shuxin Wang[1], Nan Cheng[4], Zhen Wang[3], Xianqin Jiang[1], Xuqun Zheng[1], Wenjing Xu[1], Minyan Ye[1], Longwei Zhao[5], Meiyun Wen[5], Jianlou Niu[1], Weijian Sun[2], Pingping Shen[2, 3*], Zhifeng Huang[1, *], Xiaokun Li [1, *]

Affiliations

[1]Oujiang Laboratory (Zhejiang Lab for Regenerative Medicine, Vision, and Brain Health), State Key Laboratory of Macromolecular Drugs and Large-scale Preparation, School of Pharmaceutical Sciences, Wenzhou Medical University, Wenzhou, Zhejiang 325035, China

[2] Department of Colorectal Surgery, The First Affiliated Hospital of Wenzhou Medical University, Wenzhou, 325027, China

[3]State Key Laboratory of Pharmaceutical Biotechnology and Clinical Stem Cell Center, the Affiliated Drum Tower Hospital of Nanjing University Medical School, School of Life Sciences, Nanjing University, Nanjing, 210023, China

[4]School of Integrative Medicine, Nanjing University of Chinese Medicine, Nanjing, 210023, China

[5]Department of Pharmacology, School of Basic Medical Sciences, Wenzhou Medical University, 325035 Wenzhou, Zhejiang, China

***Correspondence：** xiaokunli@wmu.edu.cn (X.L.), hzf@wmu.edu.cn (Z.H.), ppshen@nju.edu.cn (P.S)

[†]These authors contributed equally to this work

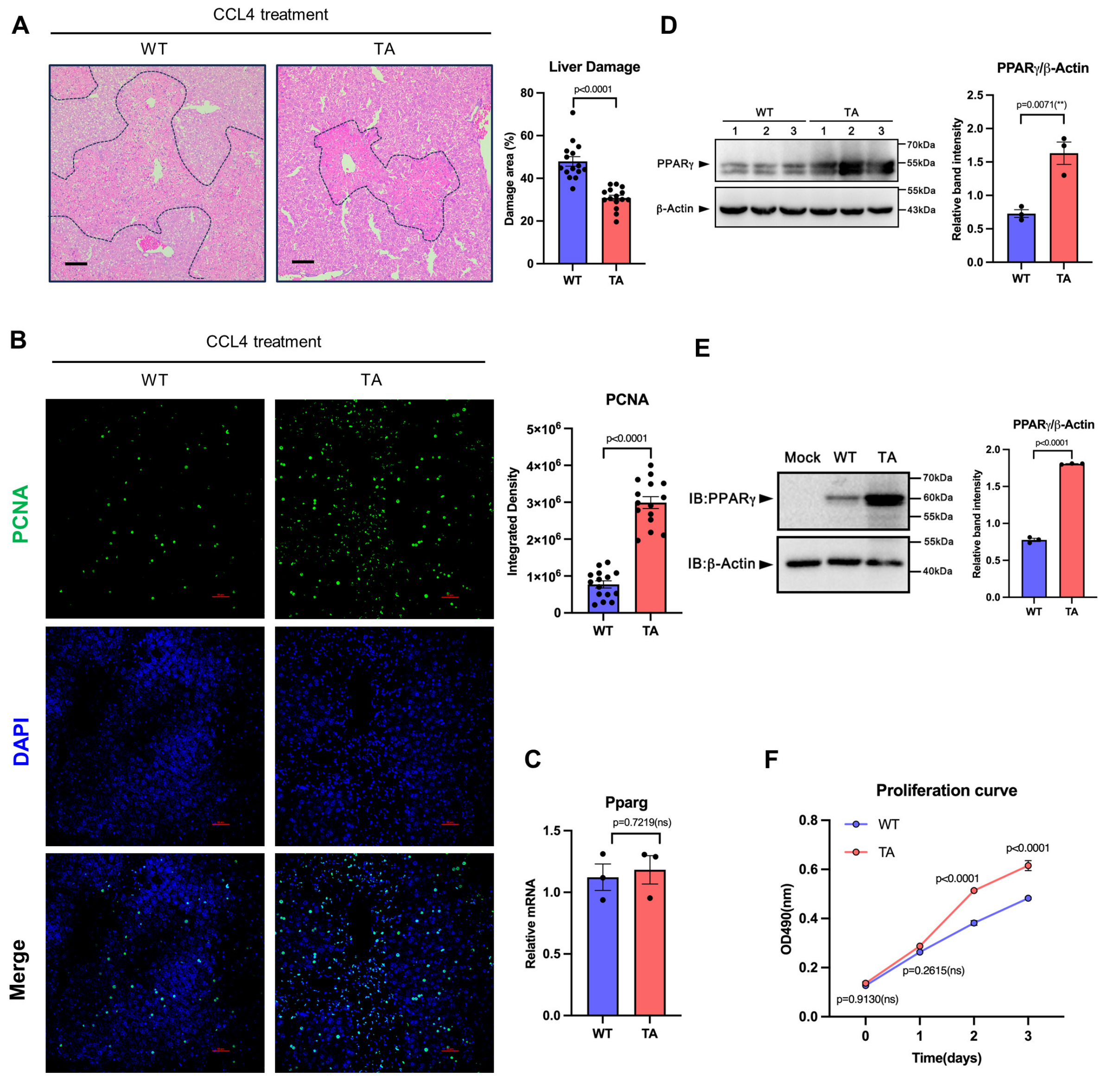

Figure S1. T166A promotes hepatocyte proliferation and accelerates liver regeneration.

(A) WT and T166A (TA) mice were treated with CCL4. After 48h treatment, the mice were euthanasia, the liver tissues were applied to the experiments. H&E staining of liver tissues (n=3). 100×magnification, scale bar, 100μm. Each H&E slide is captured in five fields of view, and the liver injury area in each field of view is calculated to determine the average injury area. Every point in the statistical chart represents the injury area percentage in each field of view.

(B) Confocal microscopy analysis of PCNA protein positive cells in liver tissues (n=3). 200×magnification, scale bar, 50μm. Each slide is captured in five fields of view, and the integrated density (IntDen) was measured by Image J software. Every point in the statistical chart represents the IntDen value in each field of view.

(C) Q-PCR analysis the mRNA levels of PPARγ in liver tissues (n=3).

(D) Western blotting analysis of endogenous PPARγ protein levels in in liver tissues (n=3). Every lane contains total protein from one mouse. Every point in the statistical chart represents the band signal intensity of one lane.

(E) AML-12 cells were transfected with PPARγ2 plasmid. Western blotting analysis of ectopic PPARγ protein levels in cells. Experiments were repeated three times.

(F) The growth curves of WT and TA overexpressed AML-12 cells were measured by CCK-8 assay (n=3).

Data were analyzed by two-way ANOVA followed by Bonferroni's test (**F**) or Student's t-test (**A-E**). Data are presented as mean ± SEM. $^{*}P < 0.05$. $^{**}P < 0.01$, $^{***}P < 0.001$.

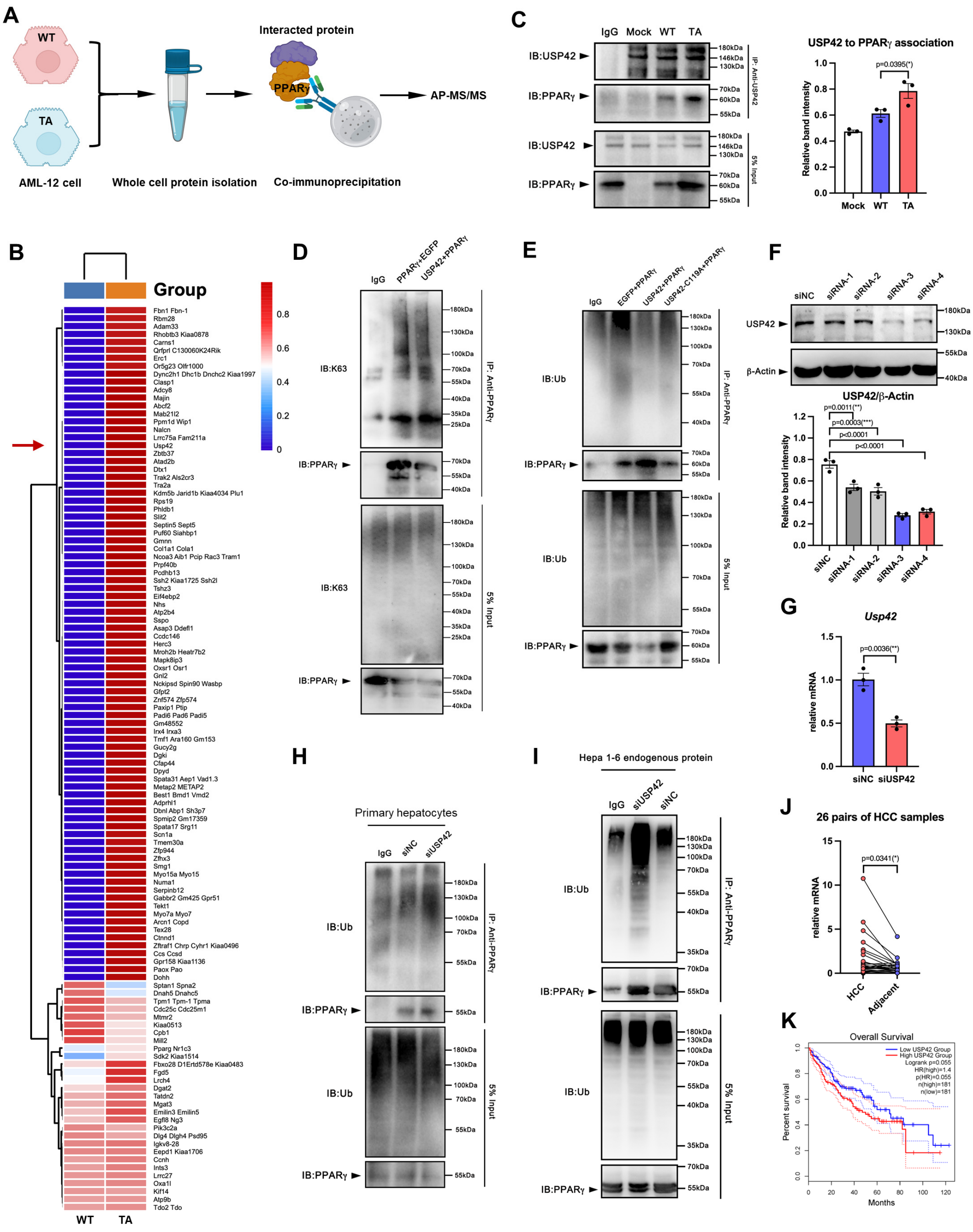

Figure S2. Identification of USP42 is a novel DUB of PPARγ in hepatocytes.

(A) Schematic of the experiment. The whole cell lysis of WT and TA overexpressed AML-12 cells were subjected to Co-IP and AP-MS/MS.

(B) Heatmap of identified protein-protein interactions (PPIs) in WT and TA group. The score was calculated by MiST algorithm (mass spectrometry interaction statistics).

(C) HEK293T cells were co-transfected USP42 and WT or TA PPARγ. The interactions between proteins were analyzed by Co-IP. Experiments were repeated three times.

(D) K63-linked ubiquitination assay of PPARγ in HEK293T cells co-transfected with WT PPARγ, EGFP and USP42 and treated with 10μM MG-132 for 12 h.

(E) HEK293T cells were transfected with WT PPARγ and WT USP42 or C119A USP42 plasmids. Cells were treated with 10μM MG-132 for 12 h. Total ubiquitination assay of PPARγ in HEK293T cells.

(F) Hepa1-6 cells were transfected with siRNA for USP42 knockdown. Western blotting analysis of the knockdown efficiency of four siRNA sequences. Experiments were repeated three times.

(G) siRNA-4 was selected to knock down the USP42 in Hepa1-6 and the mRNA level of USP42 was evaluated by Q-PCR (n=3).

(H) Primary hepatocytes were transfected with siRNA-4, and the total ubiquitination of PPARγ was analyzed.

(I) Hepa1-6 cells were transfected with siRNA-4, and the total ubiquitination of PPARγ was analyzed.

(J) cDNA chip analysis of the human samples from 26 HCC patients (n=26).

(K) TCGA database analysis of the survival of USP42 high or low expression HCC patients (n=181).

Data were analyzed by one-way ANOVA followed by Tukey's test (**C**, **F**) or Student's t-test (**G, J**). Data are presented as mean ± SEM. $^{*}P < 0.05$. $^{**}P < 0.01$, $^{***}P < 0.001$.

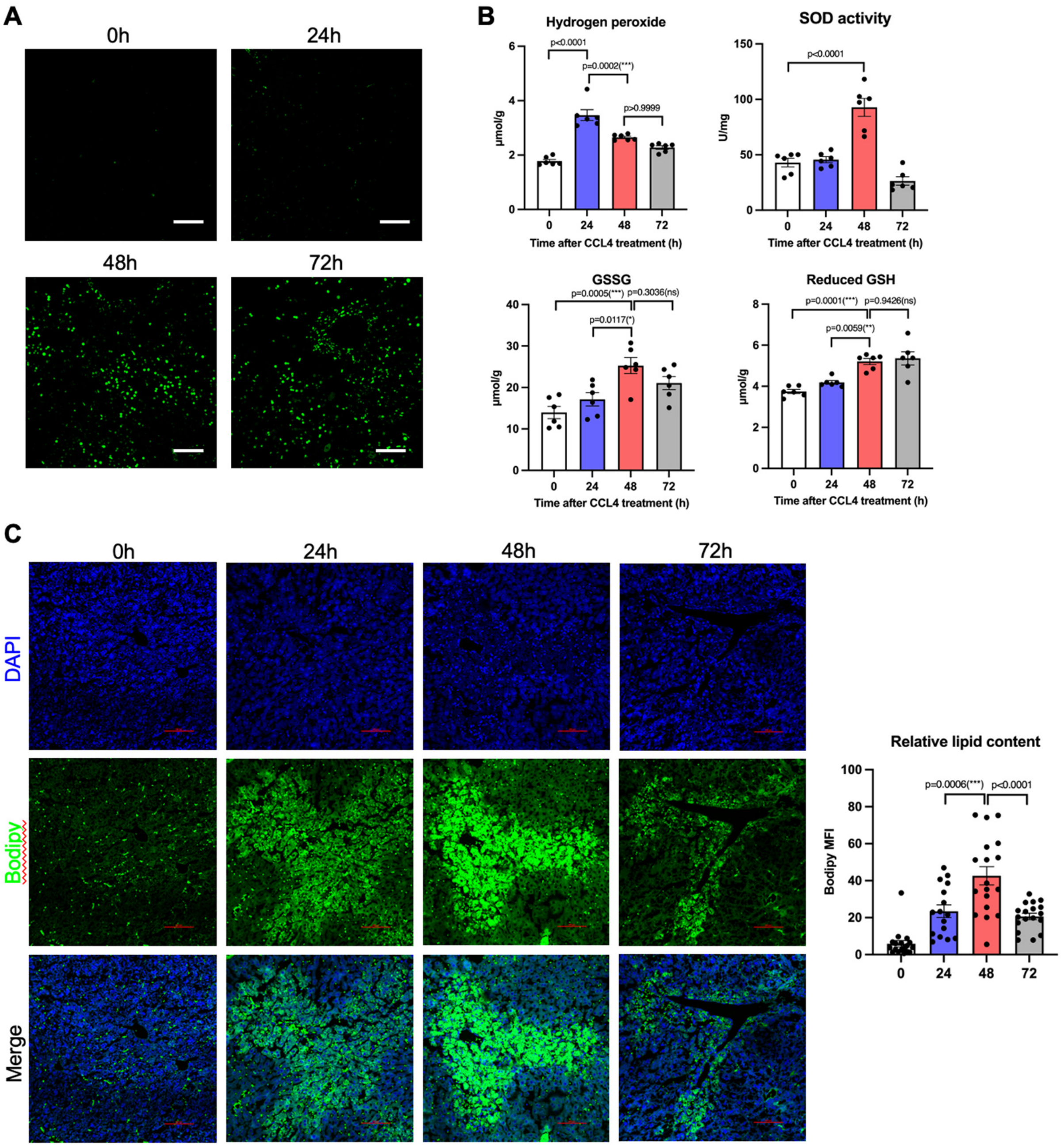

Figure S3. USP42 and PPARγ protein levels are correlated with redox state in liver tissues.

(A) Confocal microscopy analysis of PCNA protein positive cells in liver tissues (n=6).

(B) The contents of hydrogen peroxides, SOD activity, GSSG and reduced GSH in the liver tissue was measured using the commercial kits (n=6).

(C) Confocal microscopy analysis of the lipid droplets (Biodipy) in liver tissues (n=6). 200× magnification; scale bar, 100μm. Each slide is captured in three fields of view, and the integrated density (IntDen) was measured by Image J software. Every point in the statistical chart represents the IntDen value in each field of view.

Data were analyzed by one-way ANOVA followed by Tukey's test (**B-C**). Data are presented as mean ± SEM. $^{*}P < 0.05$. $^{**}P < 0.01$, $^{***}P < 0.001$.

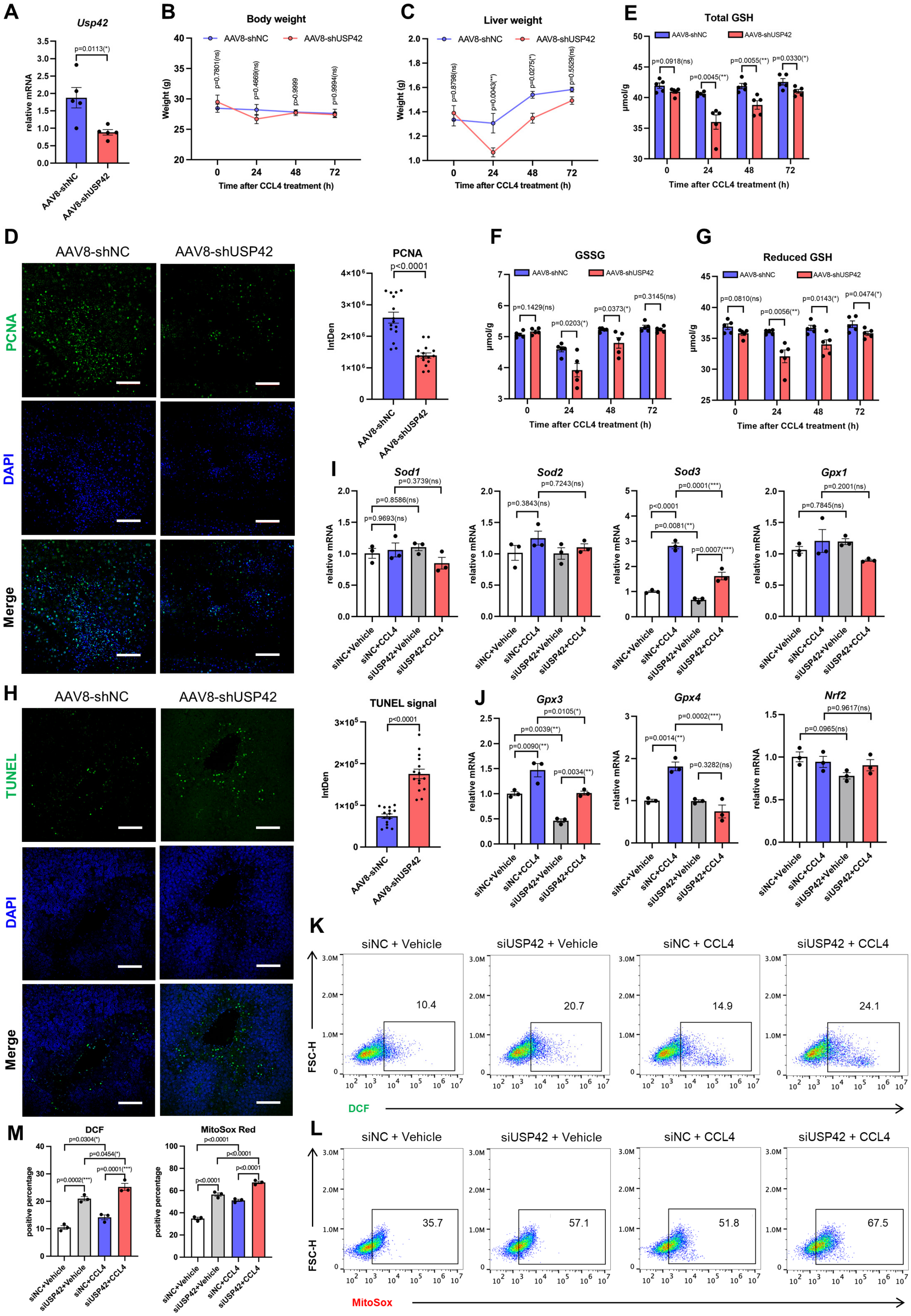

Figure S4. USP42 knockdown exacerbates hepatocyte apoptosis and oxidation stress.

(A) Male C57BL/6J mice were injected with AAV8-shNC or AAV8-shUSP42 via tail vein. After three weeks, the mRNA levels of USP42 in liver tissues were evaluated by Q-PCR (n=5).
(B-C) The body weight and liver weight in every time points (n=5).
(D) Confocal microscopy analysis of PCNA protein positive cells in liver tissues of 48h time point (n=5). 200×magnification, scale bar, 100μm. Each slide is captured in three fields of view, and the integrated density (IntDen) was measured by Image J software. Every point in the statistical chart represents the IntDen value in each field of view.
(E-G) The contents of total GSH, GSSG and reduced GSH in the liver tissue was measured using the commercial kits (n=5).
(H) Confocal microscopy analysis of TUNEL positive cells in liver tissues of 48h time point (n=5). 200×magnification, scale bar, 100μm. Each slide is captured in four fields of view, and the integrated density (IntDen) was measured by Image J software. Every point in the statistical chart represents the IntDen value in each field of view.
(I-J) Q-PCR analysis the mRNA levels of GPX1, GPX3, GPX4, SOD1, SOD2, SOD3 and Nrf2 in cultured primary hepatocytes treated with siRNA and CCL4 (n=5).
(K-L) Primary hepatocytes were transfected with siNC or siUSP42 followed by the CCL4 treatment *in vitro*. The total ROS content (K) and mitochondrial ROS (L) were measured by DCF and MitoSox probe in flow cytometry.
(M) The statistical chart related to (K-L) (n=3).
Data were analyzed by one-way ANOVA followed by Tukey's test (**E-G, I-J, M**) or two-way ANOVA followed by Bonferroni's test (**B-C**) or Student's t-test (**A, D, H**). Data are presented as mean ± SEM. $^{*}P < 0.05$. $^{**}P < 0.01$, $^{***}P < 0.001$.

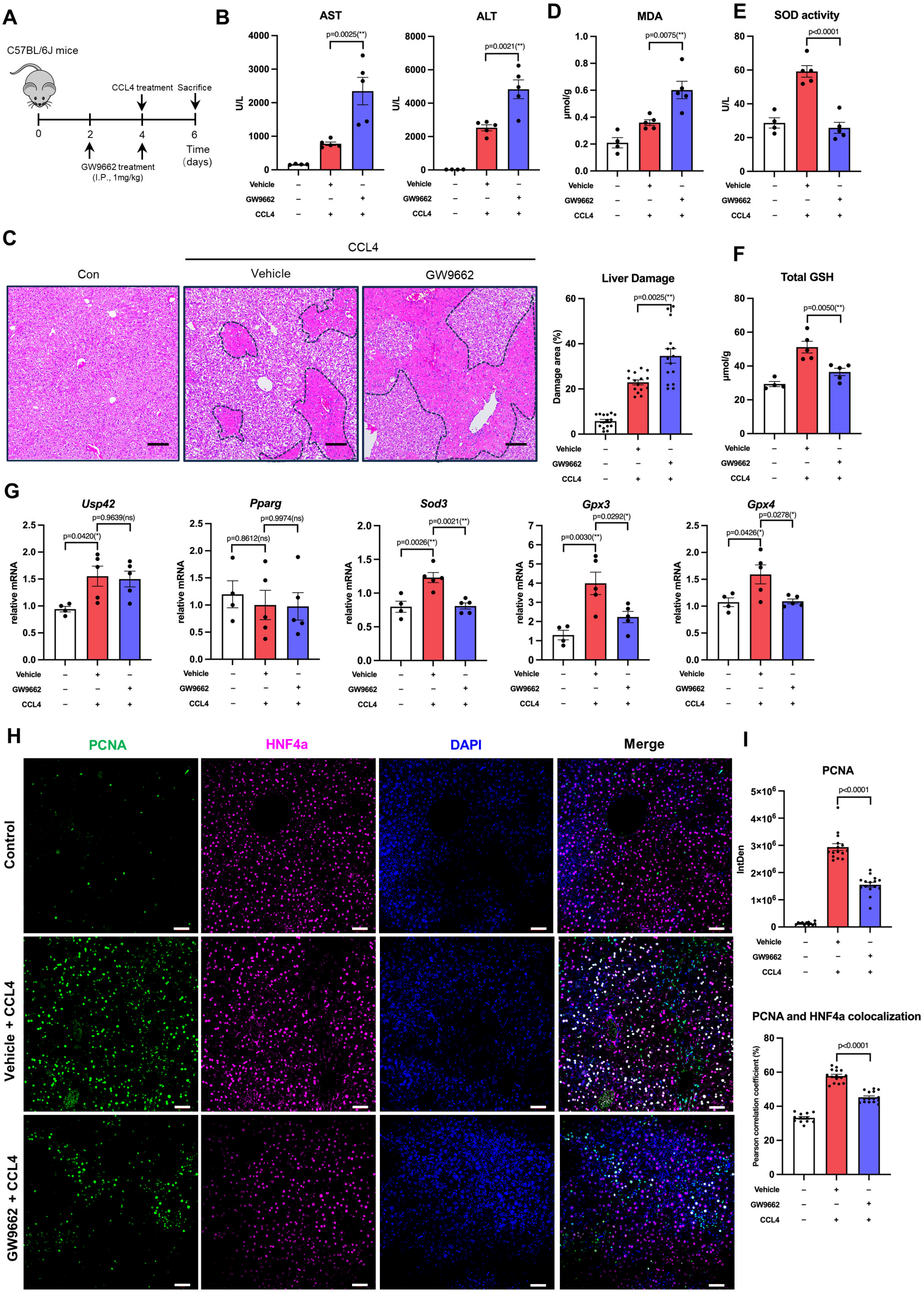

Figure S5. PPARγ antagonism significantly hinders the liver repair.

(A) Schematic of the experiment. Male C57BL/6J mice were treated with GW9662 (1mg/kg) at day 2. At day 4, mice were subjected to CCL4 treatment and GW9662 (1mg/kg) administration. After 48h, the mice were euthanasia, the serum and liver tissues were applied to the experiments.

(B) The activity of ALT and AST was measured by using the fully automated dry biochemical analyzer NX700i (FUJIFILM) (n=5).

(C) H&E staining of liver tissues (n=5). 100×magnification, scale bar, 100μm. Each H&E slide is captured in three fields of view, and the liver injury area in each field of view is calculated to determine the average injury area. Every point in the statistical chart represents the injury area percentage in each field of view.

(D-F) The MDA content (D), the SOD activity (E) and the total GSH level (F) in the liver tissue was measured using the commercial kits (n=5).

(G) Q-PCR analysis of the mRNA levels of Usp42, Pparg, Sod3, Gpx3 and Gpx4 in liver tissues (n=5).

(H-I) Confocal microscopy analysis of PCNA and HNF4α protein positive cells in liver tissues (n=6). 200×magnification, scale bar, 50μm (H). Each slide is captured in three fields of view, and the integrated density (IntDen) and was the pearson correlation coefficient measured by Image J software. Every point in the statistical chart represents the IntDen value or the pearson correlation coefficient in each field of view (I).

Data were analyzed by one-way ANOVA followed by Tukey's test (B-G and I). Data are presented as mean ± SEM. $^{*}P < 0.05$. $^{**}P < 0.01$, $^{***}P < 0.001$.

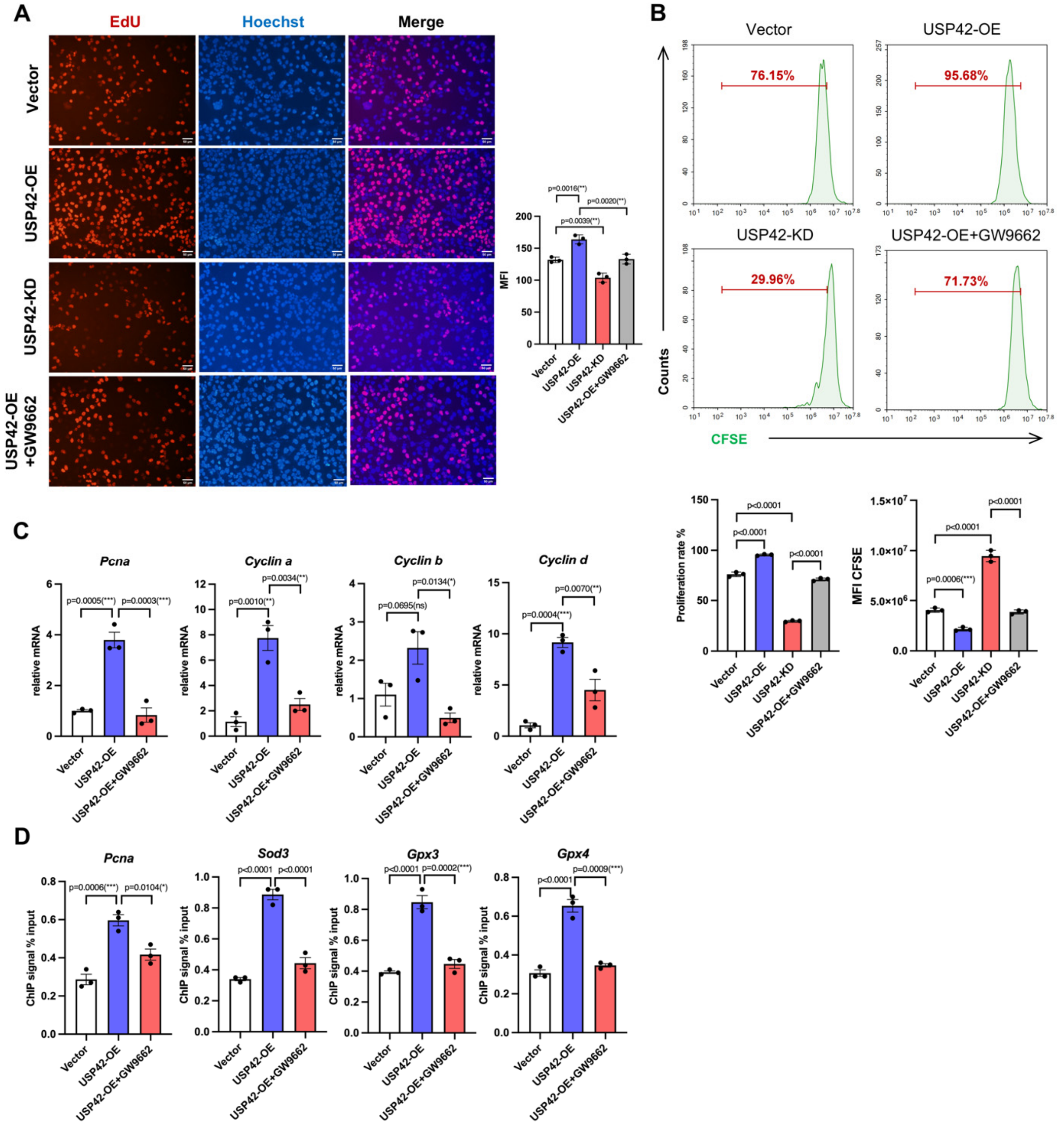

Figure S6. USP42 controls PPARγ transcriptional action on proliferation and redox balance.

(A) 5-ethynyl-2-deoxyuridine (EdU) staining of the Hepa1-6 cell lines and the cell proliferation was calculated by quantification of the fluorescence intensity of EdU. 200×magnification, scale bar, 50μm. Each group included three replicate wells, and the average fluorescence intensity for each well was calculated. Each point in the statistical chart represents the average fluorescence intensity of a single well (n=3).

(B) CFSE assay analysis of the proliferative activity of USP42-OE, USP42-KD and USP42-OE+GW9662(n=3).

(C) Q-PCR analysis of the mRNA levels of Pcna, Cyclin a, Cyclin b and Cyclin d in Hepa1-6 cell lines (n=3).

(D) ChIP-Q-PCR analysis of the relative binding affinity of PPARγ on the promotors of Pcna, Sod3, Gpx3/4 in constructed Hepa1-6 cell lines (n=3).

Data were analyzed by one-way ANOVA followed by Tukey's test (**A-D**). Data are presented as mean ± SEM. $^{*}P < 0.05$. $^{**}P < 0.01$, $^{***}P < 0.001$.

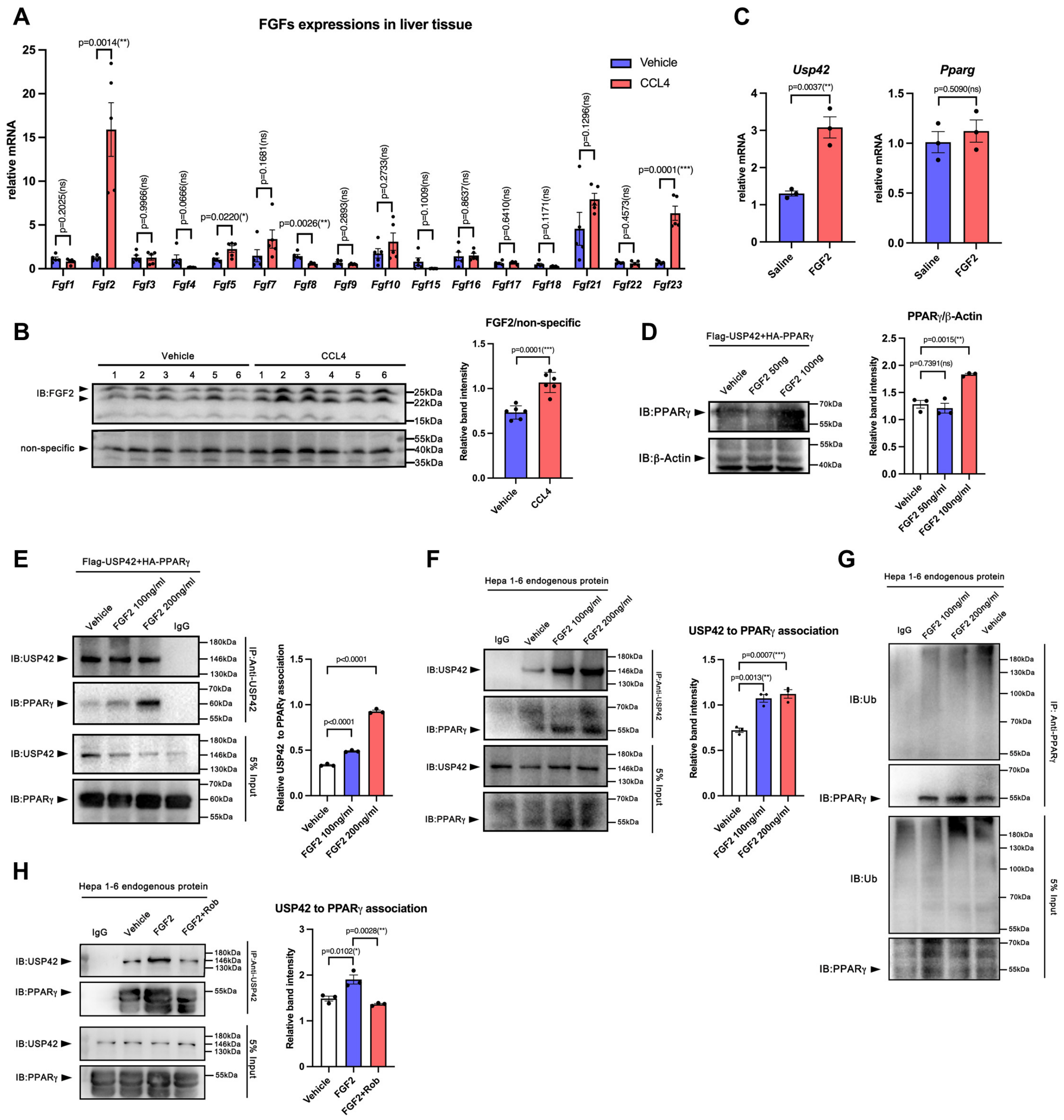

Figure S7. FGF2 can enhance the USP42-PPARγ interaction.

(A) Male C57BL/6J mice were treated with CCL4 for 48h. The mRNA levels of FGFs in liver tissues were analyzed by Q-PCR (n=5).

(B) Male C57BL/6J mice were treated with CCL4 for 48h. The protein levels of FGF2 in liver tissues were analyzed by Western blotting (n=6). Every lane contains total protein from one mouse. Every point in the statistical chart represents the band signal intensity of one lane.

(C) Hepa1-6 cells were treated with 100ng/ml recombined mouse FGF2 protein for 48h. Q-PCR analysis of the USP42 and PPARγ gene expressions (n=3).

(D) HEK293T cells were co-transfected with USP42 and PPARγ plasmids. After 24h transfection, recombined mouse FGF2 protein was added into culture system (50ng/ml and 100ng/ml) for treatment of 48h. Western blotting analysis of the PPARγ protein levels. Experiments were repeated three times.

(E) Co-IP analysis of the interaction between USP42 and PPARγ upon the FGF2 administration in HEK293T cells. Experiments were repeated three times.

(F) Co-IP analysis of the interaction between endogenous USP42 and PPARγ upon FGF2 administration in Hepa1-6 cells. Experiments were repeated three times.

(G) Ubiquitination assay of endogenous PPARγ in Hepa1-6 cells upon FGF2 administration.

(H) Hepa1-6 cells were treated vehicle or Roblitinib (Rob) upon the FGF2 administration. Co-IP analysis of the interaction between endogenous USP42 and PPARγ. Experiments were repeated three times.

Data were analyzed by one-way ANOVA followed by Tukey's test (**D-F, H**) or two-way ANOVA followed by Bonferroni's test (**A**) or Student's t-test (**B-C**). Data are presented as mean ± SEM. $^{*}P < 0.05$. $^{**}P < 0.01$, $^{***}P < 0.001$.

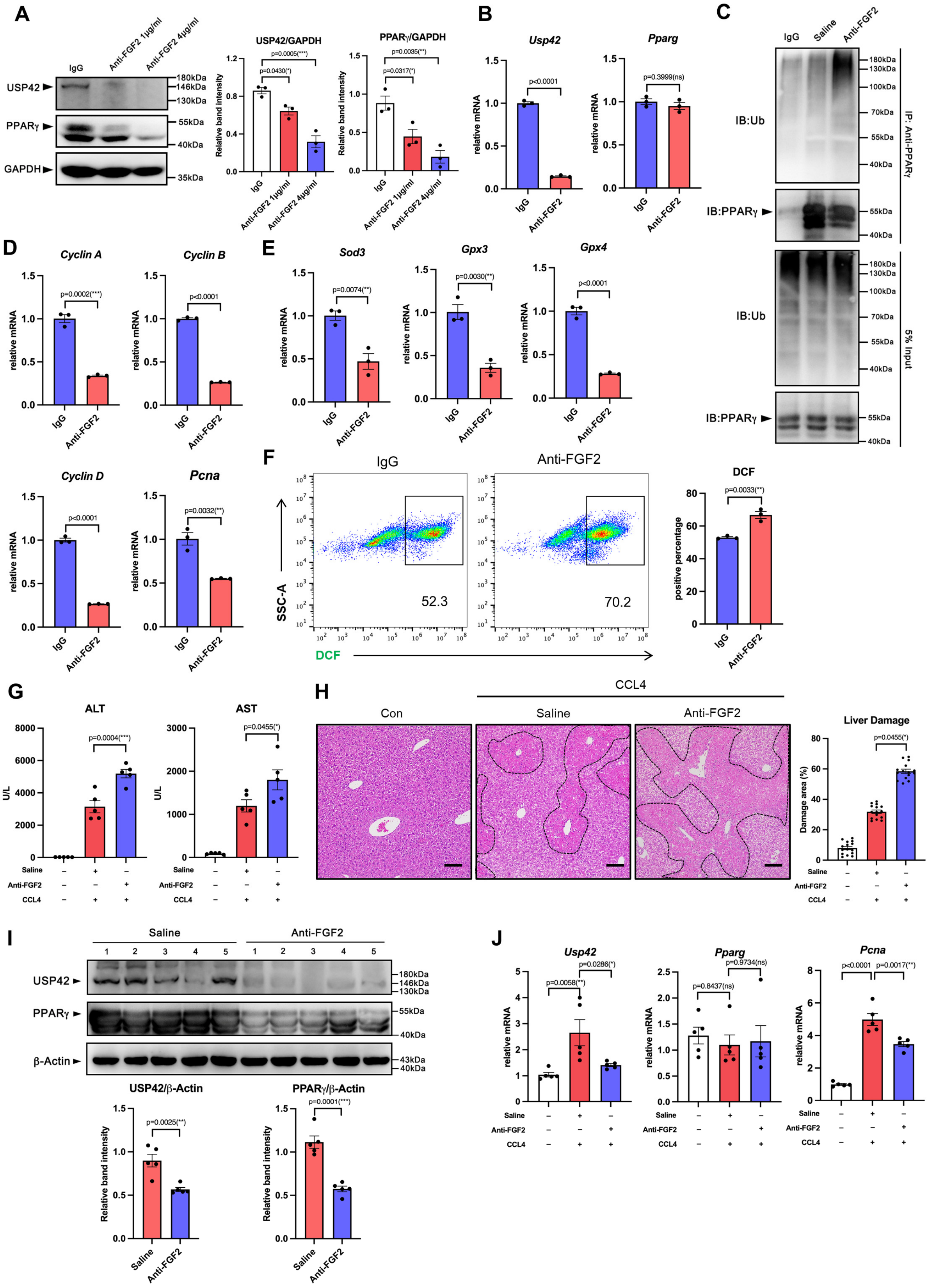

Figure S8. Endogenous FGF2 neutralization inhibits the USP42-PPARγ axis and impedes the liver regeneration.

(A) Hepa1-6 cells were treated with anti-FGF2 neutralization antibody (1µg/ml or 4µg/ml) or isotype IgG for 36h. Western blotting was performed for detection of the USP42 and PPARγ protein levels. Experiments were repeated three times.

(B) Hepa1-6 cells were treated with anti-FGF2 neutralization antibody (4µg/ml) or isotype IgG for 36h. Q-PCR analysis of the gene expression levels of *Usp42* and *Pparg* (n=3).

(C) Ubiquitination assay of endogenous PPARγ in Hepa1-6 cells upon anti-FGF2 antibody administration.

(D-E) Hepa1-6 cells were treated with anti-FGF2 neutralization antibody (4µg/ml) or isotype IgG for 36h. Q-PCR analysis of the gene expression levels of proliferative genes (D) and the anti-ROS genes (E) (n=3).

(F) Hepa1-6 cells were treated with anti-FGF2 neutralization antibody (4µg/ml) or isotype IgG for 36h followed by 500µM CuOOH treatment for 4h. The total ROS content was measured by DCF probe in flow cytometry.

(G) Male C57BL/6J mice were treated with anti-FGF2 neutralization antibody (20µg/mouse) once daily for three consecutive days. Then, mice were subjected to CCL4 treatment. After 48h, the mice were euthanasia, the serum and liver tissues were applied to the experiments. The activity of ALT and AST was measured by using the fully automated dry biochemical analyzer NX700i (FUJIFILM) (n=5).

(H) H&E staining of liver tissues (n=5). 100×magnification, scale bar, 100µm. Each H&E slide is captured in three fields of view, and the liver injury area in each field of view is calculated to determine the average injury area. Every point in the statistical chart represents the injury area percentage in each field of view.

(I) Isolated liver tissues were homogenized. The whole tissue protein was analyzed by western blotting to evaluate the levels of USP42 and PPARγ (n=5). Every lane contains total protein from one mouse. Every point in the statistical chart represents the band signal intensity of one lane.

(J) Q-PCR analysis the mRNA levels of *Usp42, Pparg* and *Pcna* in liver tissues (n=5).

Data were analyzed by Student's t-test (**A-B, D-F**) or one-way ANOVA followed by Tukey's test (**G-J**). Data are presented as mean ± SEM. $^{*}P < 0.05$. $^{**}P < 0.01$, $^{***}P < 0.001$.

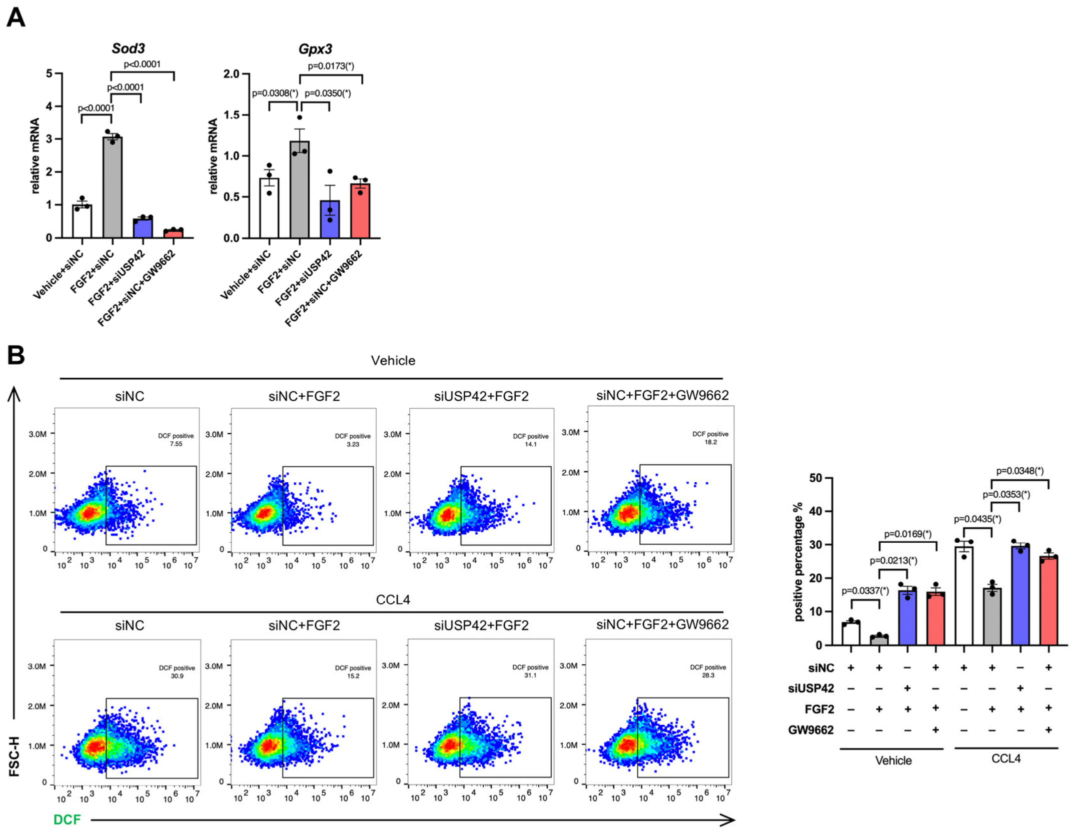

Figure S9. USP42 knockdown or PPARγ antagonism inhibits FGF2 mediated anti-ROS function.

(A) Hepa1-6 cells were transfected with siNC or siUSP42 with or without GW9662 treatment for 48h. Then, cells were treated with 500μM CuOOH for 4h. Q-PCR analysis of Sod and Gpx3 gene expressions (n=3).

(B) Hepa1-6 cells were transfected with siNC or siUSP42 with or without GW9662 treatment for 48h. Then, cells were treated with 500μM CuOOH for 4h. The total ROS content was measured by commercial kit (n=3). Data were analyzed by one-way ANOVA followed by Tukey's test (**A-B**). Data are presented as mean ± SEM. $^{*}P < 0.05$. $^{**}P < 0.01$, $^{***}P < 0.001$.

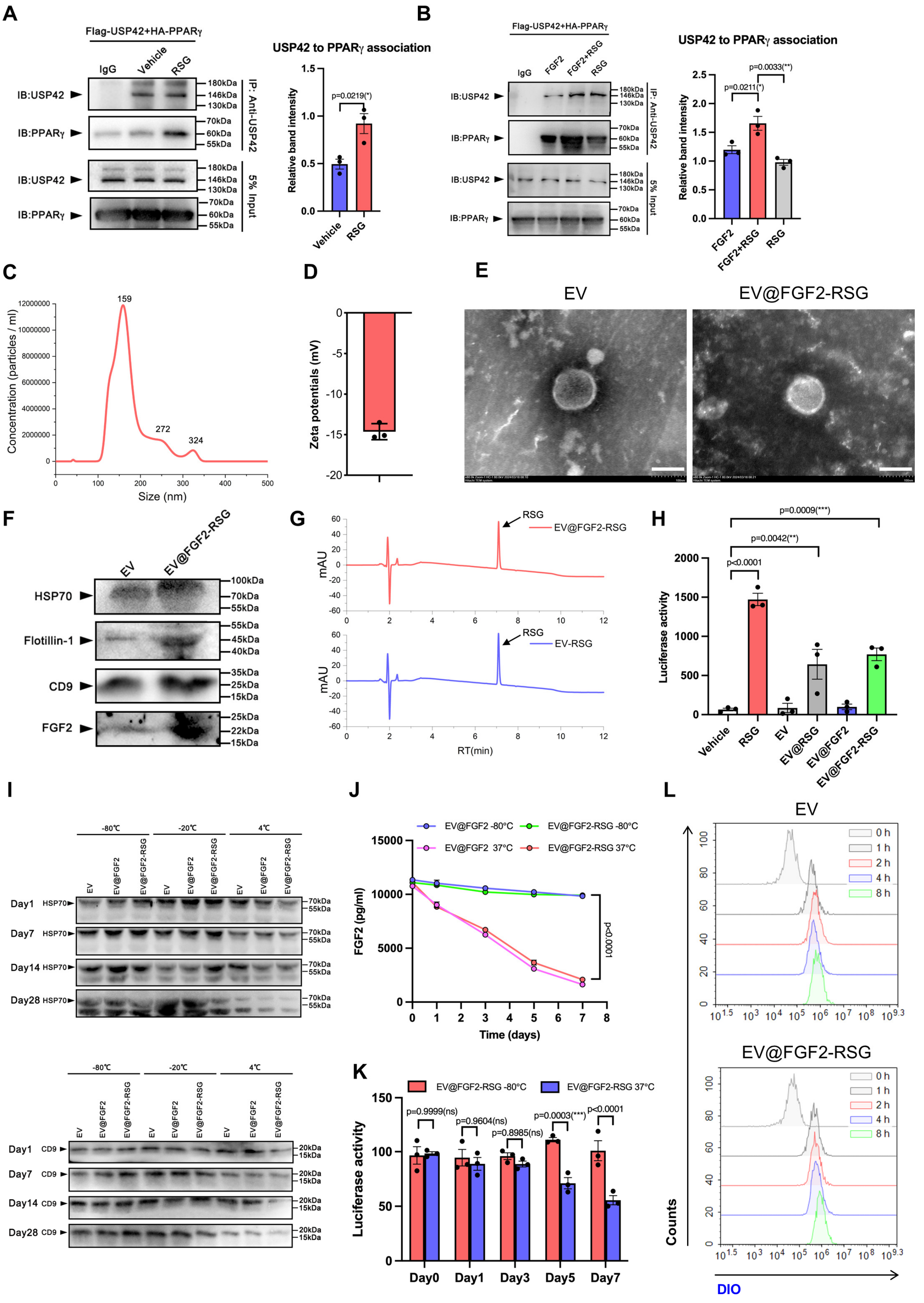

Figure S10. Design, preparation and characterization of the EV@FGF2-RSG.

(A) HEK293T cells were co-transfected with USP42 and PPARγ plasmids for 24h. Cells were then treated with 100nM RSG. Co-IP analysis of the interactions of USP42 and PPARγ. Experiments were repeated three times.

(B) HEK293T cells were co-transfected with USP42 and PPARγ plasmids for 24h. Cells were then treated with 100nM RSG or 200ng/ml FGF2. Co-IP analysis of the interactions of USP42 and PPARγ. Experiments were repeated three times.

(C) Nanoparticle tracking analysis (NTA) of EV@FGF2-RSG from UC-MSC.

(D) The zeta potential of EV@FGF2-RSG.

(E) Representative image of EV@FGF2-RSG and control empty EV as observed by transmission electron microscopy (TEM), scale bar, 100nm.

(F) Western blotting analysis of the relative protein levels of EV-associated markers (HSP70, Flotillin-1 and CD9) and FGF2 protein in EV@FGF2-RSG and empty EV.

(G) The drug loading capacity of EV@FGF2-RSG and EV@RSG was determined by high performance liquid chromatography (HPLC).

(H) Luciferase reporter assay evaluating the transcriptional activity of wild type PPARγ upon the treatment with RSG, EV, EV@RSG, EV@FGF2, EV@FGF2-RSG (n=3).

(I) Western blotting analysis of the relative protein levels of EV-associated markers (HSP70 and CD9) in EV@FGF2, EV@FGF2-RSG and empty EV stored at -80℃, -20℃ or 4℃ for 0, 7, 14, and 28 days.

(J) ELISA detection of the FGF2 content in EV@FGF2 and EV@FGF2-RSG stored at -80℃ or 37℃ for 1, 3, 5 and 7 days (n=3).

(K) Luciferase reporter assay evaluating the transcriptional activity of wild type PPARγ upon the treatment with EV@FGF2-RSG that stored at -80℃ or 37℃ for 0-7 days (n=3).

(L) Internalization of EV@FGF2-RSG and empty EV by primary hepatocytes was detected by flow cytometry.

Data were analyzed by Student's t-test (**A-B**) or one-way ANOVA followed by Tukey's test (**H-I, K**) or two-way ANOVA followed by Bonferroni's test (**J**). Data are presented as mean ± SEM. $^{*}P < 0.05$. $^{**}P < 0.01$, $^{***}P < 0.001$.

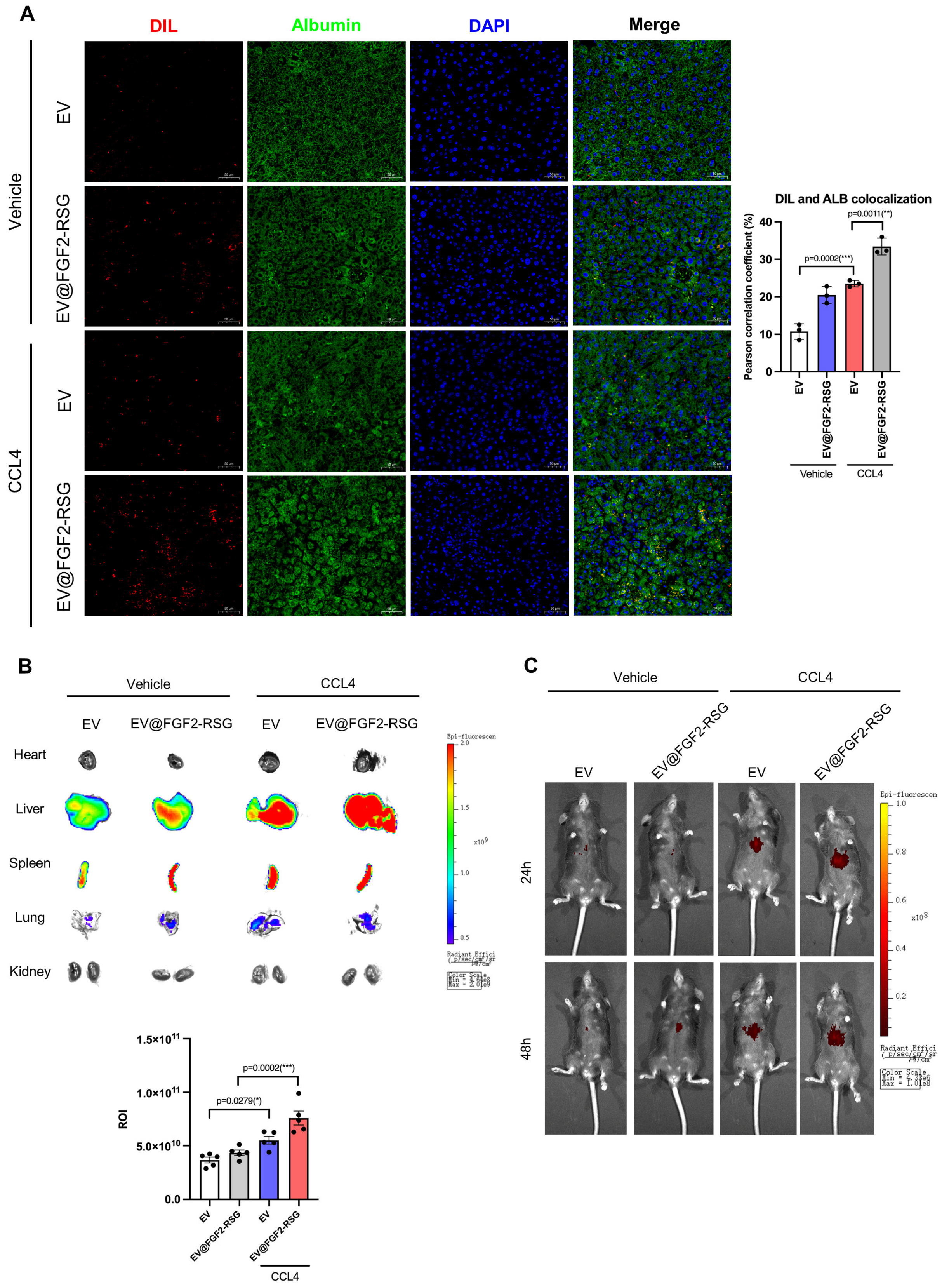

Figure S11. Liver targeting efficiency detection of EV@FGF2-RSG.

(A) For analysis of the hepatocyte uptake, mice were injected intravenously with DIL-labeled EV@FGF2-RSG. Frozen liver sections were immunostaining of the Albumin, and the confocal microscopy was

performed to analyze the colocalization of DIL-labeled EV@FGF2-RSG and Albumin. 200×magnification, scale bar, 50μm. (n=3)

(B) Biodistribution of DIR-labelled EV@FGF2-RSG in normal and CCL4-induced acute liver injury female mice (n=3).

(C) Imaging of fluorescence intensity of indicated organs. Region of interest (ROI) analysis of fluorescence intensity of organs.

Data were analyzed by one-way ANOVA followed by Tukey's test (**A-B**). Data are presented as mean ± SEM. $^{*}P < 0.05$. $^{**}P < 0.01$, $^{***}P < 0.001$.

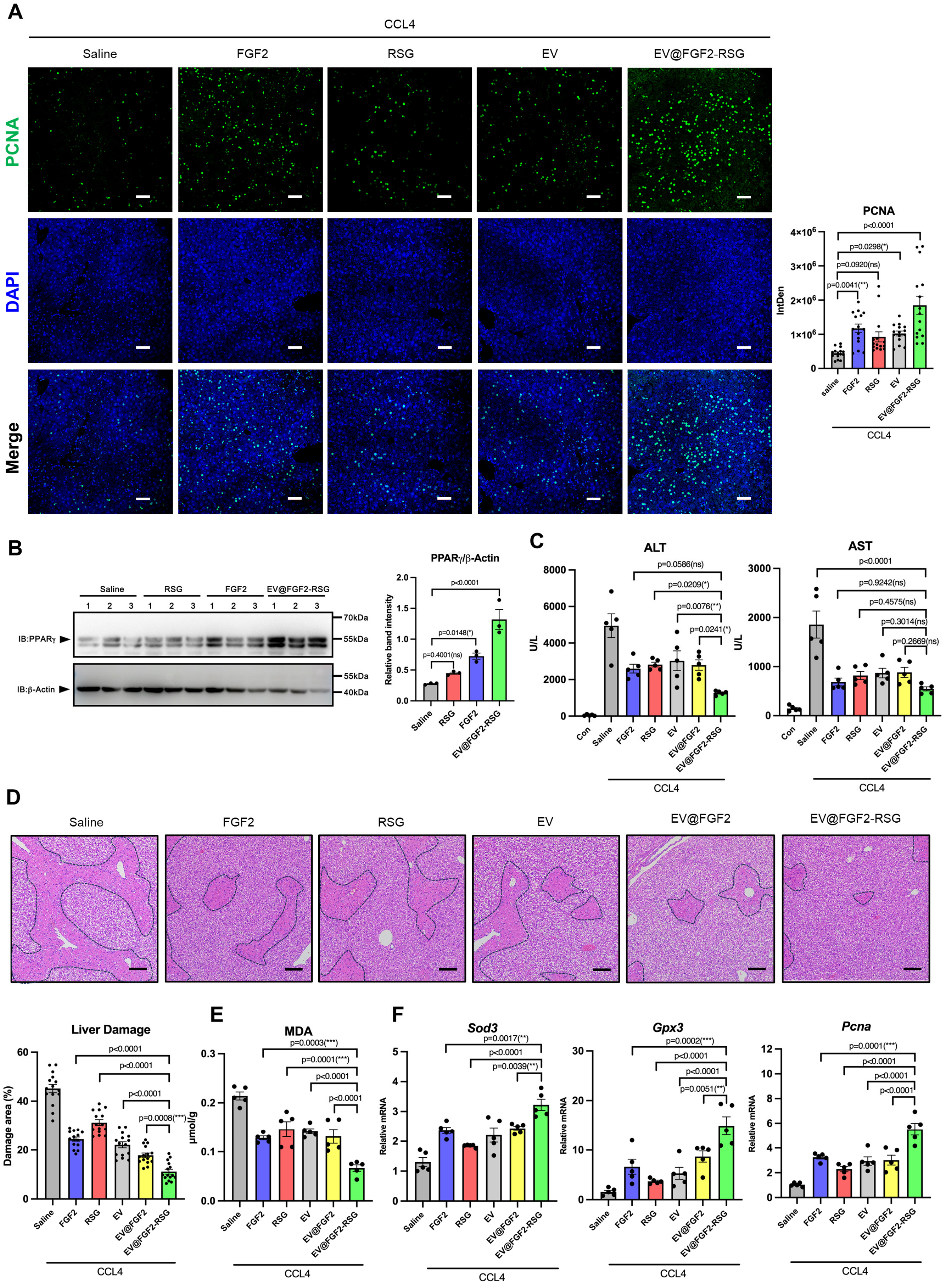

Figure S12. RSG enhanced the efficacy of FGF2 on PPARγ increase and liver regeneration.

(A) Confocal microscopy analysis of PCNA protein positive cells in liver tissues (n=5). 200×magnification, scale bar, 50μm. Each slide is captured in three fields of view, and the integrated density (IntDen) was

measured by Image J software. Every point in the statistical chart represents the IntDen value in each field of view.

(B) Western blotting analysis of PPARγ protein levels in liver tissues (n=5). Every lane contains total protein from one or two mouse. Every point in the statistical chart represents the band signal intensity of one lane.

(C) The EV@FGF2-RSG (5mg/kg) or EV@FGF2 and the control empty EV (5mg/kg) were injected into mice via tail vein after 2h CCL4 treatment. The RSG (5mg/kg) and FGF2 (0.5mg/kg) were set as the positive control. The activity of ALT and AST was measured by using the fully automated dry biochemical analyzer NX700i (FUJIFILM) (n=5).

(D) H&E staining of liver tissues (n=5). 100×magnification, scale bar, 100μm. Each H&E slide is captured in three fields of view, and the liver injury area in each field of view is calculated to determine the average injury area. Every point in the statistical chart represents the injury area percentage in each field of view.

(E) The MDA content in the liver tissue was measured using the commercial kits (n=5).

(F) Q-PCR analysis the mRNA levels of *Sod3, Gpx3* and *Pcna* in liver tissues (n=5).

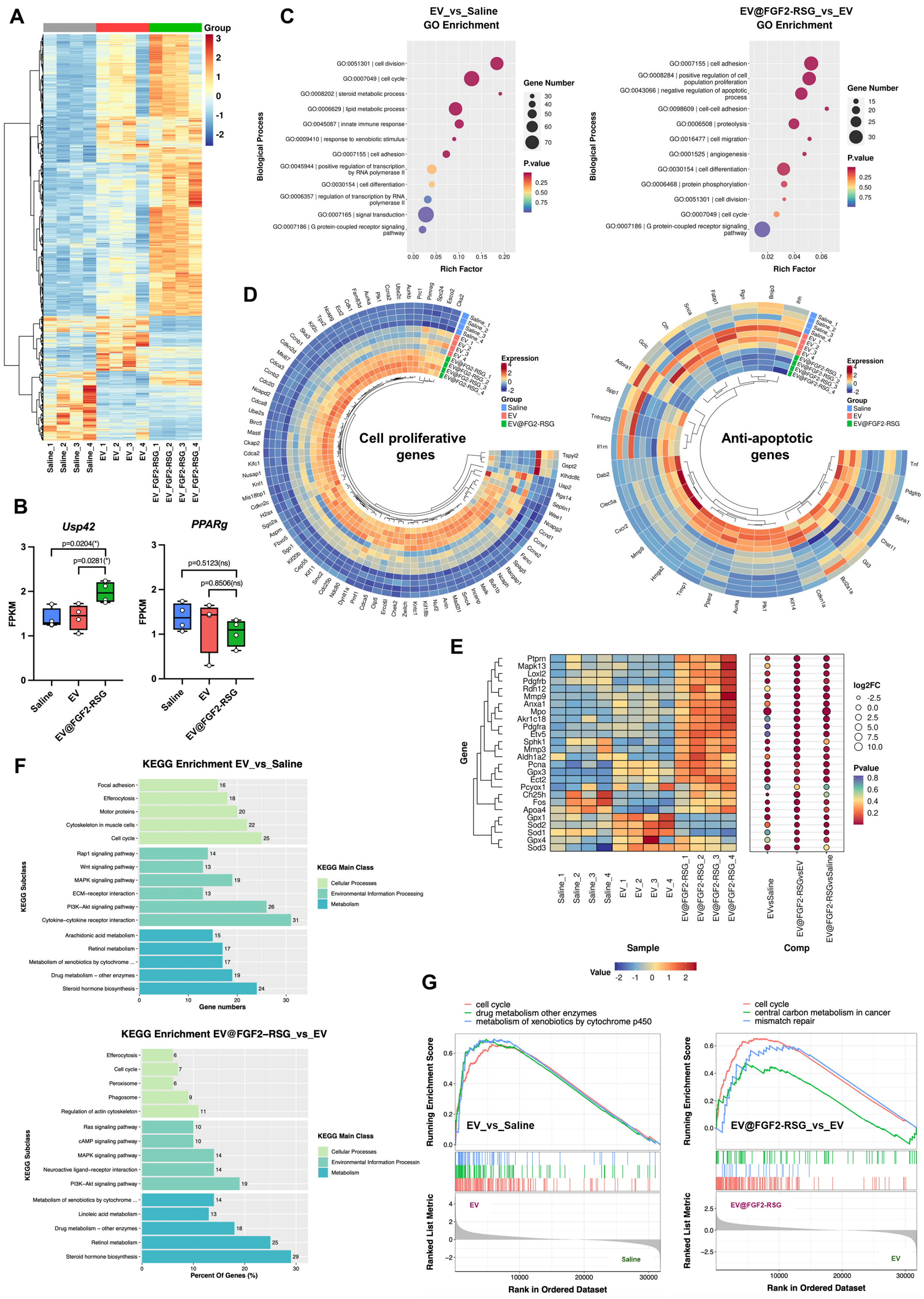

Figure S13. EV@FGF2-RSG activates the proliferation and anti-apoptotic pathways

(A) The EV@FGF2-RSG (5mg/kg), the control empty EV (5mg/kg) or the Saline control was injected into mice via tail vein after 2h CCL4 treatment. After 48h, the liver tissues were collected and subjected to RNA-Seq. Heat map of all differentially expressed genes in the three groups (n=4).

(B) The gene expressions (FPKM value) of *Usp42* and *Pparg* (n=4).

(C) GO analysis of differentially expressed genes between EV and Saline, or between EV@FGF2-RSG and EV (n=4).

(D) Heat map of cell proliferative genes and anti-apoptotic genes clustered in GO analysis among the three groups (n=4).

(E) Heat map of differentially expressed genes of redox balance among the three groups (n=4).

(F) KEGG analysis of differentially expressed genes between EV and Saline, or between EV@FGF2-RSG and EV (n=4).

(G) GSEA analysis of differentially expressed genes (n=4).

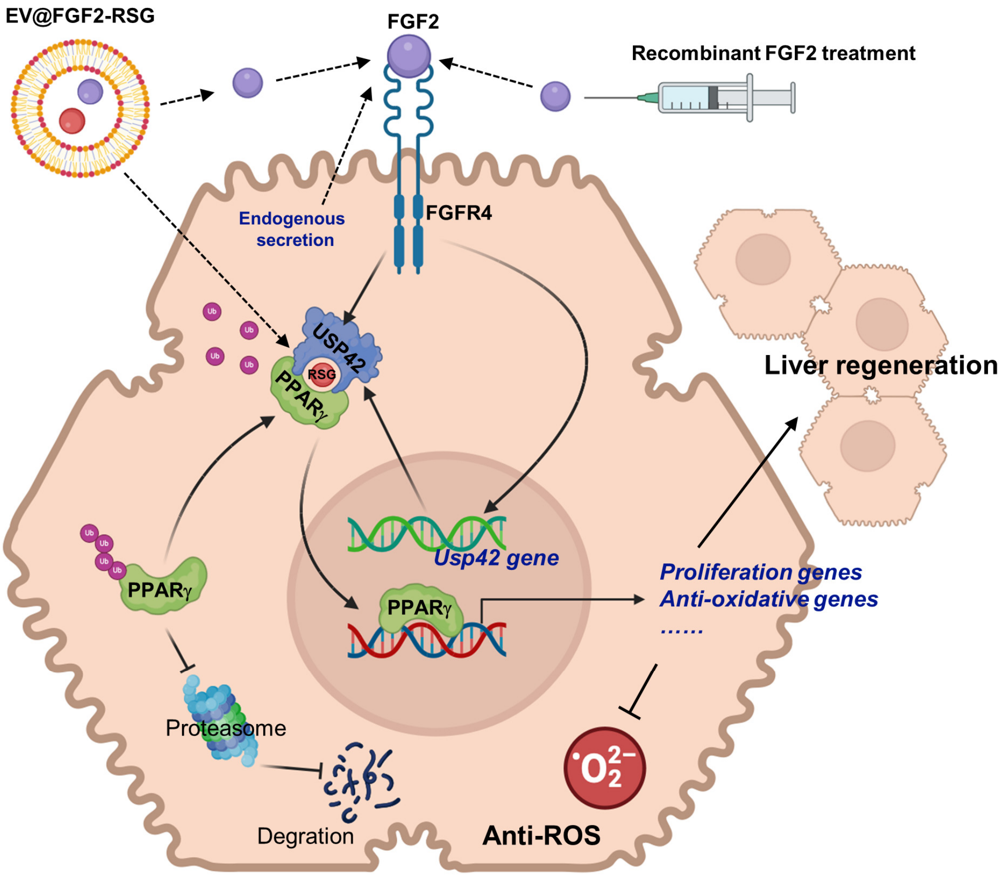

Graphical Abstract. FGF2 mediated USP42-PPARγ axis activation enhances the liver regeneration

(Designed and drawn by using Biorender)
